# Immunopeptidomics Mapping of *Listeria monocytogenes* T Cell Epitopes in Mice

**DOI:** 10.1016/j.mcpro.2024.100829

**Published:** 2024-08-13

**Authors:** Adillah Gul, Lecia L. Pewe, Patrick Willems, Rupert Mayer, Fabien Thery, Caroline Asselman, Ilke Aernout, Rein Verbeke, Denzel Eggermont, Laura Van Moortel, Ellen Upton, Yifeng Zhang, Katie Boucher, Laia Miret-Casals, Hans Demol, Stefaan C. De Smedt, Ine Lentacker, Lilliana Radoshevich, John T. Harty, Francis Impens

**Affiliations:** 1 VIB-UGent Center for Medical Biotechnology, VIB, Ghent, Belgium; 2 Department of Biomolecular Medicine, Ghent University, Ghent, Belgium; 3 Department of Pathology, University of Iowa-Carver College of Medicine, Iowa City, Iowa, USA; 4 VIB-UGent Center for Plant Systems Biology, VIB, Ghent, Belgium; 5 Department of Plant Biotechnology and Bioinformatics, Ghent University, Ghent, Belgium; 6 VIB Proteomics Core, VIB, Ghent, Belgium; 7 Center for Medical Genetics, Ghent University Hospital, Ghent, Belgium; 8 Ghent Research Group on Nanomedicines, Ghent University, Ghent, Belgium; 9 Cancer Research Institute Ghent (CRIG), Ghent, Belgium; 10 Department of Microbiology and Immunology, University of Iowa-Carver College of Medicine, Iowa City, Iowa, USA; 11 Interdisciplinary Graduate Program in Immunology, University of Iowa, Iowa City, Iowa, USA; 12 Department of Immunology and Genomic Medicine, National Jewish Health, Denver, Colorado, USA

**Keywords:** epitope discovery, mass spectrometry, *Listeria monocytogenes*, bacterial infection, MHC

## Abstract

*Listeria monocytogenes* is a foodborne intracellular bacterial model pathogen. Protective immunity against *Listeria* depends on an effective CD8^+^ T cell response, but very few T cell epitopes are known in mice as a common animal infection model for listeriosis. To identify epitopes, we screened for *Listeria* immunopeptides presented in the spleen of infected mice by mass spectrometry–based immunopeptidomics. We mapped more than 6000 mouse self-peptides presented on MHC class I molecules, including 12 high confident *Listeria* peptides from 12 different bacterial proteins. Bacterial immunopeptides with confirmed fragmentation spectra were further tested for their potential to activate CD8^+^ T cells, revealing VTYNYINI from the putative cell wall surface anchor family protein LMON_0576 as a novel *bona fide* peptide epitope. The epitope showed high biological potency in a prime boost model and can be used as a research tool to probe CD8^+^ T cell responses in the mouse models of *Listeria* infection. Together, our results demonstrate the power of immunopeptidomics for bacterial antigen identification.

## Highlights

• Identification of >6000 MHC class I immunopeptides in *Listeria*-infected mice.
• High-confident detection of 12 *Listeria*-derived immunopeptides.
• Discovery of VTYNYINI as a potent *Listeria* CD8^+^ T cell epitope.

## Graphical Abstract

**Figure:**
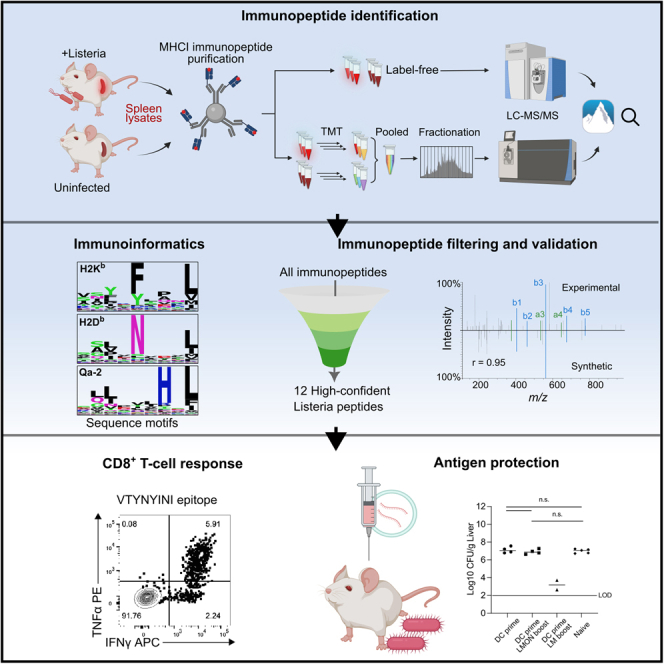

## In Brief

Immunopeptidomics is a key technology for bacterial epitope and antigen discovery. This study describes the identification of 12 bacterial immunopeptides in the spleen of mice infected with *Listeria monocytogenes*, an intracellular bacterial model pathogen. Among these, VTYNYINI from the cell wall surface protein LMON_0576 was capable of activating CD8+ T cells. This novel epitope showed high biological potency in a prime-boost model and serves as a valuable research tool to monitor CD8+ T cell responses in *Listeria* infection models.

*Listeria monocytogenes* (referred to as *Listeria*) is a foodborne pathogen and causative agent of human listeriosis, a rare but potentially severe disease in pregnant women, elderly and immunocompromised individuals (1). *Listeria* poses serious challenges for the food industry due to its ability to persist and thrive in various environmental stress conditions including broad ranges of temperature, pH, and salinity (2). Upon ingestion, *Listeria* can cross the intestinal barrier and enter the bloodstream from where it can disseminate to the liver and spleen (3). Moreover, *Listeria* has the ability to cross the blood-brain and fetoplacental barriers causing severe complications (4). As a facultative intracellular pathogen, *Listeria* can evade the humoral immune system by hiding inside host cells. *Listeria* therefore triggers its own uptake by binding to host membrane proteins leading to receptor-mediated endocytosis (5). Once inside the internalization vacuole, *Listeria* actively secretes the pore-forming toxin listeriolysin O (LLO) and phospholipases to rupture the vesicle membrane and gain access to the host cell cytosol (6). From there, it can replicate and further spread to neighboring cells by actin-based motility (1).

Intracellular bacteria like *Listeria* possess an astonishing ability to reside and replicate within host cells, posing unique challenges to the immune system often resulting in chronic or persistent infections that are more difficult to treat with antibiotics (7, 8). In addition, the overuse of antibiotics along with social and economic factors has increased the spread of antimicrobial resistance (AMR) in many bacteria (9), including *Listeria* (10). AMR takes a serious toll worldwide, estimated to be responsible for five million deaths in 2019 (11). Consequently, the World Health Organization has declared AMR as one of the most imminent health threats facing humanity (12). To combat drug resistance, the development of novel antibacterial vaccines regained attention as an effective and long-lasting strategy in recent years (13, 14). Traditional antibacterial vaccines have been and are routinely implemented in public health care systems, successfully protecting against diphtheria, pertussis, meningococcus, tetanus, and many others (15). However, limitations in conventional vaccine approaches and the global COVID-19 pandemic led to the development and implementation of new vaccine approaches such as viral vectors and mRNA vaccines. Compared to conventional vaccine platforms, mRNA vaccines are simple to design, fast to produce, safe to administer, and effective in generating CD8^+^ T cell responses (16, 17). Together, these features make mRNA vaccines attractive to orchestrate rapid and precise responses against infectious diseases, including intracellular bacterial infections.

Unlike for extracellular bacteria, there is a paucity of vaccines against clinically relevant intracellular bacteria, with the exception of antibacterial vaccines for tuberculosis and typhoid fever (15). Intracellular bacteria present a clinical challenge as they evade humoral immune responses and interfere with host cell antigen presentation, making it particularly challenging to formulate antigen candidates and develop vaccines. Therefore, a mechanistic understanding of how intracellular pathogens manipulate and evade the host immune system is crucial for the development of effective treatments and preventive measures (18). While commercial interest in a *Listeria* vaccine is limited today, *Listeria* serves as an excellent model for intracellular bacterial infections and vaccine studies (19, 20). Since the 1960s, *Listeria* has been widely used as a model pathogen to understand host–bacteria interactions both in cell and animal systems. Given its ease to culture, facile manipulation, relative safety (*e.g.*, compared to problematic bacteria such as *Mycobacteria*), and application in mouse models, *Listeria* remains widely adopted by immunologists and microbiologists (1). For instance, the murine model of listeriosis is the most commonly used to investigate host defense against intracellular pathogens, particularly CD8^+^ T cell biology (19, 21). The immune clearance of intracellular *Listeria* depends on CD8^+^ T cell–mediated cytotoxic responses; therefore, an effective vaccine should be capable of eliciting strong cellular immunity (22). A crucial prerequisite for such vaccine development is elucidating the repertoire of epitopes and antigens presented on infected cells (23); however, this knowledge is generally lacking.

In recent years, mass spectrometry (MS)–based immunopeptidomics has emerged as an analytical technology for detecting bacteria-derived immunopeptides (23). Contemporary MS instruments and data analysis algorithms enable the identification of dozens of bacterial immunopeptides in a single analysis (24, 25, 26). Despite these technological innovations, there is a lack of studies exploring the *Listeria* immunopeptidome using MS, especially in mice. In the Immune Epitope Database (IEDB) (27), only 118 epitopes from 13 *Listeria* antigens are listed for murine hosts. Of these, 51 epitopes presented on MHC class I molecules and the majority are derived from two highly abundant secreted *Listeria* proteins LLO (25 peptides) and p60 (10 peptides). Identification of additional epitopes could greatly aid immune and vaccine studies using mouse infection models of *Listeria*. In the present study, we therefore employed an immunopeptidomics workflow to screen for *Listeria* peptides presented on MHC class I molecules in the spleen of infected mice. Among more than 6000 mouse self-peptides, we identified 12 *Listeria* immunopeptides from 12 different bacterial antigens. To our knowledge, none of these putative epitopes were previously reported, and screening selected peptides for their ability to stimulate CD8^+^ T cells revealed a *bona fide* epitope from the putative cell wall surface anchor family protein LMON_0576. Immunization with this epitope did not protect animals against a challenge infection, but it can be used as a research tool to probe CD8^+^ T cell responses in the mouse models of *Listeria* infection.

## Experimental Procedures

### *Listeria* Infection of C57BL/6 Mice

All animal studies and procedures were approved by the University of Iowa Animal Care and Use Committee (IACUC), under U.S. Public Health Service assurance, Office of Laboratory Animal Welfare guidelines with IACUC approval numbers #3051102 and #2032090. For the initial screen, C57BL/6 mice were infected intravenously via tail veil injection between 8 and 12 weeks of age with *L. monocytogenes* strain EGD at 4.1 × 10^6^ colony forming units (CFUs) per animal (or saline control for uninfected). Mice were assessed daily after inoculation and euthanized if they lost more than 25% of their body weight, became moribund, or dropped below a body condition score equal or less than two. Mice were sacrificed 72 h following infection and spleens were harvested for MHC class I immunoprecipitation.

### Generation of Immunoaffinity Columns for MHC Class I Pull Down

M1/42.3.9.8 (#BE0077, BioXcell, referred to as M1/42) monoclonal antibody reacts against mouse H-2 MHC class I alloantigen (all haplotypes). To prepare immunoaffinity columns, 1 ml of resuspended protein Recombinant Protein G - Sepharose 4B beads (#101242, Invitrogen) were washed with 100 mM Tris pH 8.0 before 3 mg of purified antibody was added and incubated at room temperature for 1 h on a rolling tube mixer device. M1/42 antibody bound-beads were washed with 0.2 M sodium borate pH 9.0 (#B3545, Sigma-Aldrich, Merck) and crosslinked with fresh 20 mM dimethylpimelimidate (#D8388, Sigma-Aldrich, Merck) dissolved in sodium borate solution for 30 min on a rolling tube mixer device. After crosslinking, antibody–beads complexes were washed with 0.2 M ethanol amine pH 8.0 (#149582500, Thermo Fisher Scientific) to quench the crosslinking reaction.

### Tissue Lysis

Mouse spleens were lysed with a mild lysis buffer containing 1% (v/v) octyl-β,D-glucopyranoside (#O9882, Sigma-Aldrich, Merck), 0.25% (v/v) sodium deoxycholate (#1065040250, Millipore, Merck), 1.25x cOmplete protease inhibitor cocktail (#4693159001, Roche), 1 mM phenylmethylsulfonyl fluoride (#52332, Sigma-Aldrich, Merck), 0.2 mM iodoacetamide (#I1149-5G, Sigma-Aldrich, Merck), and 1 mM ethylendiamine tetraacetic acid (#EDS, Sigma-Aldrich, Merck) in Ca/Mg-free PBS (#14190-169, Thermo Fisher Scientific). Ice cold lysis buffer was added at a ratio of 1 ml per 0.1 g of frozen tissue and the tissue was lysed using an Ultra Turrax mixing homogenizer. The lysate was cleared by centrifugation at 2000*g* for 10 min at 4 °C and the supernatant was further cleared by a second centrifugation step at 16,100*g* for 35 min at 4 °C.

### Isolation and Purification of Immunopeptides

Prior to immunoprecipitation, Econo glass columns (#7374150, Bio-Rad) were cleaned with ethanol and Milli-Q water. Per sample, 100 μl settled protein A sepharose beads were added to the Econo glass column and washed with 100 mM Tris pH 8.0, as described (28). Cleared lysate was added to the beads and rotated at 4 °C for 1 h to deplete endogenous antibodies. Antibody-depleted lysate was retrieved and incubated with M1/42 immunoaffinity columns overnight at 4 °C on a rolling tube mixer device. Beads were washed with ice-cold solutions in the cold room: twice with 150 mM sodium chloride in 20 mM Tris pH 8.0, twice with 400 mM NaCl in 20 mM Tris pH 8.0, again twice with 150 mM NaCl in 20 mM Tris pH 8.0, and finally twice with 20 mM TRIS pH 8.0. MHC class I:peptide complexes were eluted by applying 5 ml 10% acetic acid per 1 ml settled beads. Eluted immunopeptides were purified using C18 ODS 100 mg SampliQ columns (#5982-1111, Agilent Technologies) using a vacuum manifold. After initial loading to the C18 column, samples were re-loaded four times for a total of five loads. C18 columns were washed with 1 ml 2% acetonitrile (ACN) (#1000292500, Sigma-Aldrich, Merck) in 0.2% acetic acid prior to the elution of MHC class I peptides by applying twice 300 μl of 30% ACN in 0.1% trifluoroacetic acid (TFA), followed by pooling of the eluates and complete drying in 2  mL protein LoBind tubes (#0030108450, Eppendorf). For further purification, immunopeptides were reconstituted in 100 μl of 2% ACN in 0.2% TFA for 15 min in an ultrasonic bath. OMIX C18 pipette tips (#A57003MB, Agilent Technologies) were conditioned three times with 200 μl of 80% ACN in 0.2% TFA, followed by five times 200 μl of 0.2% TFA. Resolubilized MHC peptides were loaded onto the conditioned OMIX tips by pipetting up and down 10 times, washed with 100 μl of 0.2% TFA, and eluted by pipetting up and down 10 times with 80 μl of 30% ACN in 0.2% TFA, followed by 20 μl of 30% ACN in 0.2% TFA. Eluates were pooled and divided into two equal fractions per sample to allow parallel label-free and tandem mass tag (TMT)-labeling analysis. Both aliquots were completely dried and stored at −20 °C until further use.

### Labeling, Prefractionation, and LC-MS/MS Analysis of TMT Fractions

Dried immunopeptides were dissolved in 10 μl of 100 mM tetraethylammonium bicarbonate (#T7408-100Ml, Sigma-Aldrich, Merck) by vortexing and sonicating for 15 min. TMT16plex labels (TMTpro, 0.5 mg, #A44521, Thermo Fisher Scientific) were dissolved in 41 μl of anhydrous ACN and were regularly vortexed for 5 min to completely dissolve the labels. Next, 50 μg of TMT-label was added to each sample of peptides. Uninfected samples were labeled with the 129N, 130N, and 131N TMTpro labels, while *Listeria*-infected samples were labeled with 132N, 133N, and 134N. Peptides were incubated with the TMT-labels for 1 h at room temperature while shaking at 700 rpm. One microliter of hydroxylamine (#15675820, Fluka, Thermo Fisher Scientific) was then added to quench the reaction followed by incubation for 15 min at room temperature while shaking at 700 rpm. After quenching, the TMT-labeled samples were pooled and dried completely. The TMT-labeled and pooled immunopeptides were then separated into 12 fractions using a reversed-phase C18-column at pH 5.5. Dried peptides were solubilized in 100 μl of 2% ACN and 0.1% TFA in ultrapure water. Ninety-five microliters thereof was injected into an LC-system, consisting of a capillary pump (#G1376A, Agilent), an isocratic pump (#G1310A, Agilent), a multiple wavelength detector (#G1365B, Agilent), a column compartment (#G1316A, Agilent), a degasser (#G1379B, Agilent), and a well-plate autosampler (#G1367A, Agilent). Peptides were first loaded onto a 4 cm trapping column (made in-house, 250 μm internal diameter, 5 μm beads diameter, C18 Reprosil-HD, Dr Maisch) at a flow rate of 25 μl/min. As mobile phase, two different solvents were used. Solvent A consisted of 10 mM ammonium bicarbonate (#09830, Sigma-Aldrich, Merck) and 2% ACN in ultrapure water while solvent B consisted of 10 mM ammonium bicarbonate and 70% ACN in ultrapure water. The prefractionation started with 0% B followed by a linear increase from 0 to 100% B in 100 min between minute 20 and 120. The gradient was followed by a stationary washing phase at 100% B for 5 min and re-equilibration with 0% B for 15 min. Eluting fractions were collected using a Probot micro-fraction collector (#161403, LC-packings) into 12 MS-vials. Fractions were collected every minute from minute 20 onwards. After the first 12 fractions were collected in vials 1 to 12, the 13th fraction was again collected in vial 1 to restart the collection cycle and to pool fractions in a smart way ensuring homogenous distribution of peptide hydrophobicity within each MS vial. Fractions were collected for a total of 84 min and the fractionated samples were vacuum-dried and stored at −20 °C prior to liquid chromatography-tandem mass spectrometry (LC-MS/MS) analysis.

### LC-MS/MS Analysis of Label-Free Fractions

Purified immunopeptides for label-free analysis were redissolved in 15 μl loading solvent (0.1% TFA in water/ACN (98:2, v/v)) from which 10 μl was injected for LC-MS/MS analysis on an Ultimate 3000 RSLC nano-LC system (Thermo Fisher Scientific) in-line connected to a Q Exactive HF mass spectrometer (Thermo Fisher Scientific) equipped with a nanospray flex ion source (Thermo Fisher Scientific). Trapping was performed at 10 μl/min for 4 min in loading solvent on a 20-mm trapping column (made in-house, 100 μm internal diameter, 5 μm beads, C18 Reprosil-HD, Dr Maisch). Peptide separation after trapping was performed on a Waters NanoEase 25 cm column (100 Å pore size, 1.8 μm particles size, 75 μm inner diameter). The Ultimate 3000’s column oven was set to 50 °C. Peptides were eluted by a nonlinear gradient from 1 to 55% MS solvent B (0.1% FA in water/ACN (2:8, v/v)) over 145 min at a flow rate of 300 nl/min, followed by a 15-min washing phase plateauing at 99% MS solvent B. Re-equilibration with 99% MS solvent A (0.1% FA in water) was performed at 300 nl/min for 50 min for a total run length of 210 min. The mass spectrometer was operated in data-dependent, positive ionization mode. One MS1 scan (*m*/*z* 300–1650, AGC target 3 × 10^6^ ions, maximum ion injection time 60 ms), acquired at a resolution of 60,000 (at 200 *m*/*z*), was followed by up to 10 tandem MS scans (resolution 15,000 at 200 *m*/*z*) of the most intense ions fulfilling predefined selection criteria (AGC target 1 × 10^5^ ions, maximum ion injection time 120 ms, isolation window 1.5 Da, fixed first mass 100 *m/z*, spectrum data type: centroid, intensity threshold 8.3 × 10^3^, minimum AGC target 1.0 × 10^3^, 1 exclusion of unassigned, 4–8, >8 positively charged precursors, peptide match off, exclude isotopes on, dynamic exclusion time 12 s). The higher-energy collisional dissociation was set to 28% normalized collision energy, and the polydimethylcyclosiloxane background ion at 445.12003 Da was used for internal calibration (lock mass).

Fractionated and TMT-labeled immunopeptides were redissolved in 20 μl loading solvent from which 5 μl was injected for LC-MS/MS analysis on an Ultimate 3000 RSLC nano-LC system (Thermo Fisher Scientific) in-line connected to a Fusion Lumos mass spectrometer (Thermo Fisher Scientific). Trapping was performed as described above and peptides were again separated on a 200 cm-long micropillar array column (μPAC, PharmaFluidics) with C18-endcapped functionality. Peptides were eluted by a nonlinear gradient from 1 to 55% MS solvent B over 79 min, starting at a flow rate of 750 nl/min switching to 300 nl/min after 15 min, followed by a 11-min washing phase plateauing at 99% MS solvent B. Re-equilibration with 99% MS solvent A was performed at 300 nl/min for 10 min adding up to a total run length of 105 min. The mass spectrometer was operated in data-dependent, positive ionization mode, automatically switching between MS and MS/MS acquisition to enable a cycle time of 3 s. One MS1 scan (*m*/*z* 375–1500, AGC target 4 × 10^5^ ions, maximum ion injection time 50 ms), acquired at a resolution of 120,000 (at 200 *m*/*z*), was followed by tandem MS scans in the orbitrap (resolution 50,000 at 200 *m/z*) of the most intense ions fulfilling predefined selection criteria (AGC target 1.0 × 10^5^ ions, maximum ion injection time 86 ms, isolation window 0.9 Da, fixed first mass 100 *m*/*z*, spectrum data type: centroid, intensity threshold 50 × 10^3^, including, 2–7 positively charged precursors, peptide match off, exclude isotopes on, dynamic exclusion time 60 s). The higher-energy collisional dissociation was set to 40% normalized collision energy, and the polydimethylcyclosiloxane background ion at 445.12003 Da was used for internal calibration (lock mass).

### Data Analysis

PEAKS Studio 11.0 (29) build 20230327 (Bioinformatics Solutions Inc.) was used for database searching against the mouse reference proteome (UP000000589, 21,866 proteins) concatenated with the *L. monocytogenes* EGD proteome (UP000016703, 2850 proteins) both downloaded from UniProtKB in August 2023. The enzyme was set to unspecific, searching peptides of length 7 to 20 residues, using a precursor error tolerance of 5 ppm and fragment ion tolerance of 0.01 Da. Met oxidation was set as variable modification. In case of TMT-labeled fractions, TMT16-plex modification (+304.21 Da) of lysine and the peptide N terminus was set as fixed modification. All results were filtered at a default 1% false discovery rate (FDR) and *Listeria* peptides with greater intensity in infected samples were marked as high-confidence peptides.

Label-free quantification and TMT reporter ion quantification was performed within PEAKS Studio on default settings. For differential analysis of peptide abundance uninfected and infected samples, peptide intensity matrices were loaded in Perseus (30). After log2 transformation and missing value imputation from normal distribution (width 0.3 and down shift 2.0), a Student’s *t* test was performed to determine significant peptide abundance changes in *Listeria*-infected and uninfected samples (n = 3).

### Bioinformatics

Sequence logos were made using Logomaker (31). NetMHCpan-4.1b (32) was used for binding prediction to mouse MHC class I alleles using the online webserver (https://services.healthtech.dtu.dk/services/NetMHCpan-4.1). Unsupervised alignment and clustering of immunopeptide sequences was performed using GibbsCluster 2.0 (33) (https://services.healthtech.dtu.dk/services/GibbsCluster-2.0/) using the default settings for MHC class I peptides of different lengths. Plots were made in Python using built-in functions of seaborn (34) and matplotlib (35). DAVID webserver (https://david.ncifcrf.gov/) (36) was used for gene set enrichment analysis, specifying gene ontology-biological process, and Reactome pathway gene sets.

### Synthetic Peptide Synthesis, LC-MS/MS, and Correlation to Experimental Peptide Spectra

Synthetic peptides were synthesized at the VIB-UGent Center for Medical Biotechnology on a SYRO Multiple Peptide Synthesizer (Multisyntech) using the standard Fmoc-base solid-phase peptide synthesis. All-natural amino acids were purchased from Novabiochem, Applied Biosystems, or Iris Biotech GmbH. The synthesis of the peptides was performed using 50 mg of the Fmoc-aa-Wang resin with a loading of 0.6 mmol/g (30 μmol scale). Synthesis with double coupling steps was performed as follows: the resin was swollen in N-methyl-2-pyrrolidon (NMP) for 20 min and the Fmoc group was removed using 40% piperidine in NMP. A mixture of 4 equiv. amino acid in NMP (0.5 M), 4 equiv. PyOxim in NMP (0.5 M), and 10 equiv. *N*-diisopropylethylamine solution (Sigma Aldrich) in NMP (2 M) were added to the resin, with subsequent reaction for 40 min at room temperature. The reaction mixture was removed, and the resin was washed with NMP. Every coupling was repeated a second time. Cleavage of the peptides was performed during 2 h at RT using the following cleavage cocktail: 88% TFA, 5% triisopropylsilane, 5% phenol, and 2% H_2_O (1 ml). After the cleavage, the resin was removed by filtration and the cleavage cocktail solution containing the peptide was added to an excess of cold tert-butyl methyl ether (MTBE) to precipitate the peptide, followed by vortex and centrifugation (4′, 4000 rpm at 4 °C). The supernatant was discarded and a fresh volume of MTBE was added to repeat vortex and centrifugation. This process was repeated three times. The residual peptides were dried under vacuum and then dissolved in H_2_O/ACN to be analyzed by reversed phase high-performance liquid chromatography. The peptides were subsequently purified using semi-preparative reversed phase high-performance liquid chromatography. The purification of the peptides was performed on an ÄKTA purifier system with a UV-VIS wavelength detector (214 nm) using an EC HPLC column (NUCLEOSIL 120-10 C18, 10 μm particle size, 250 × 10 mm, Macherey-Nagel). The fractions containing the peptides were collected and analyzed by MALDI-TOF-MS. For TMTpro labeling of synthetic peptides, dried peptides were dissolved in 100 μl of 100 mM TEAB pH 8.5 and incubated with 0.5 mg TMTpro 134N label in 20 μl 100% ACN for 1h at room temperature. The reaction was quenched with 5% hydroxylamine for 15 min at room temperature. The peptide mix was acidified in 2% TFA before performing peptide clean-up with Agilent OMIX C18 100 μl tips (A57 0003100) with pre-wash using 80% ACN, 0.1% TFA; wash steps with 2% ACN, 0.1% TFA, and elution using 60% ACN, 0.1% TFA. Synthetic peptides were analyzed using similar LC-MS/MS methods as described above.

Synthetic peptides were searched against the *Listeria* EGD database with PEAKS Studio as described above. Python 3.7 was used to calculate spectral correlation including spectrum_utils (version 0.4.2) (37, 38) and pymzML (version 2.5.2) (39). Spectrum processing was performed by the annotation of fragment ion peaks for a, b, and y ions including singly and doubly charged ions. Pearson correlations were calculated on the intensities of all annotated fragment ions per spectrum. Fragment ion annotation and correlations were calculated allowing a 0.05 Da mass error tolerance.

### Immunization, T Cell Response Measurements, Challenge Studies

Mature dendritic cells (DC) for priming were generated in B6 mice injected with B16-melanoma cells expressing FLT3L as described (40, 41). Briefly, donor splenocytes from B16-FLT3L–treated mice were coated with the specified peptide for 2 h, washed, and column purified to enrich CD11c+ cells per manufacturers protocol (Miltenyi). 0.5 × 10^6^ peptide-coated DCs were injected intravenously. DC primed mice were boosted with 10e7 CFU of Δ*actA-*Δ*inlB-L. monocytogenes* i.v. or with 2 μg of mRNA lipid nanoparticle (LNP) and spleens were harvested 7 to 8 days later (40) for *in vitro* stimulation of IFN-γ and TNF cytokine production as described (41). Naïve mice or those immunized by DC prime alone (negative control), DC prime, LMON boost (positive control), or DC prime mRNA LNP boost were challenged i.v. with virulent 5 × 10^4^ *L. monocytogenes* strain 10403s at 38 days after initial priming. CFUs were detected in the spleen and liver 3 days after challenge by plating on TSB agar containing streptomycin.

### mRNA and mRNA-LNP Formulation

The gene sequence of *Listeria* EGD LMON_0576 was cloned into a pGEM4z-plasmid vector (Promega) containing a T7 promoter, 5′ and 3′ UTR of human β globulin, and a poly(A) tail and was ordered from Genscript. The protein sequence was retrieved from the Listeriomics platform, codon optimized for mouse using the IDT codon optimization tool, and confirmed by sequencing the final construct (42). For production of the *in vitro* transcribed mRNA, the plasmid was first linearized with EcoRI (Promega) and purified using the QIAquick PCR purification kit (Qiagen). The linearized plasmid was then used as templates for the *in vitro* transcription reaction using the T7 MegaScript kit, by adding an Anti-Reverse Cap Analog (Jena Bioscience) and chemically modified N1-methylpseudouridine-5′-triphosphate (Jena Bioscience) instead of the normal nucleotide, uridine. Subsequently, this capped mRNA was purified by DNase I digestion, precipitated with LiCl, and washed with 70% ethanol. Agarose gel electrophoresis was used to analyze the final mRNA product and concentrations were determined by measuring the absorbance at 260 nm. mRNAs were stored in small aliquots at −80 °C at a concentration of 1 μg/μl.

LMON_0576 mRNA was formulated in LNPs containing the ionizable lipid C12-200 using an automated T-junction device as previously described (43). Cholesterol, DSPC (1,2-distearoyl-sn-glycero-3-phosphocholine), DMG-PEG 2000 (1,2-dimyristoyl-rac-glycero-3-methoxypolyethylene glycol-2000), and αGC (alpha-galactosylceramide) were purchased from Avanti Polar Lipids, while the ionizable lipid C12-200 was purchased from Corden Pharma. Briefly, LNPs were produced by mixing an ethanol solution of C12-200, DSPC, Cholesterol, DMG-PEG, and αGC at a molar ratio of ∼50/10/38.5/1.5/0.02 respectively, with mRNA dissolved in 25 mM sodium acetate buffer of pH 4.0, to obtain LNPs with a final C12-200:mRNA weight ratio of 20:1. The resting suspension was dialyzed against a 1000-fold volume Tris buffer 9% sucrose at pH 7.4 using slide A-Lyzer dialysis cassettes with 50 molecular weight cut off (Thermo Fisher Scientific). The final product was subjected to a size and zeta potential quality control using a Malvern Zetasizer nano-ZS (Malvern Instruments Ltd). The Quant-iT RiboGreen RNA assay was used to determine mRNA encapsulation and concentration of mRNA-LNP according to the manufacturer's protocols (Thermo Fisher Scientific). In order to release and detect the encapsulated mRNA content, mRNA particles were diluted in TE buffer containing 1% (v/v) Triton X-100 (Sigma) and incubated for 10 min at 37 °C, while the free (not-encapsulated) mRNA content was directly measured after particle dilution in TE buffer. mRNA-LNP samples were stored at −20 °C before injection.

### Experimental Design and Statistical Rationale

MHC class I immunopeptides were enriched from three *Listeria*-infected and three uninfected biological replicate samples. Spleens from six to seven different mice were pooled to generate a single biological replicate sample. Purified immunopeptides from each replicate sample were split in two fractions and analyzed by LC-MS/MS in a label-free manner or after TMTpro labeling and offline pre-fractionation to enhance immunopeptide detection. For TMT labeling, uninfected samples were labeled with the 129N, 130N, and 131N TMTpro labels, while *Listeria*-infected samples were labeled with 132N, 133N, and 134N. For *Listeria*-derived immunopeptides, a higher average abundance in the three infected samples compared to the three uninfected samples (fold change infected/uninfected >1) was used as a criterium to filter for high confident peptides, while for the mouse-derived immunopeptides, a two-sided Student’s *t* test (permutation-based FDR <0.05, log2 fold change >1) was used to select for peptides that were significantly upregulated in the infected samples (Supplemental Fig. S4).

## Results

### Screening MHC Class I Peptides Presented in *Listeria*-Infected Mice

**Figure 1:**
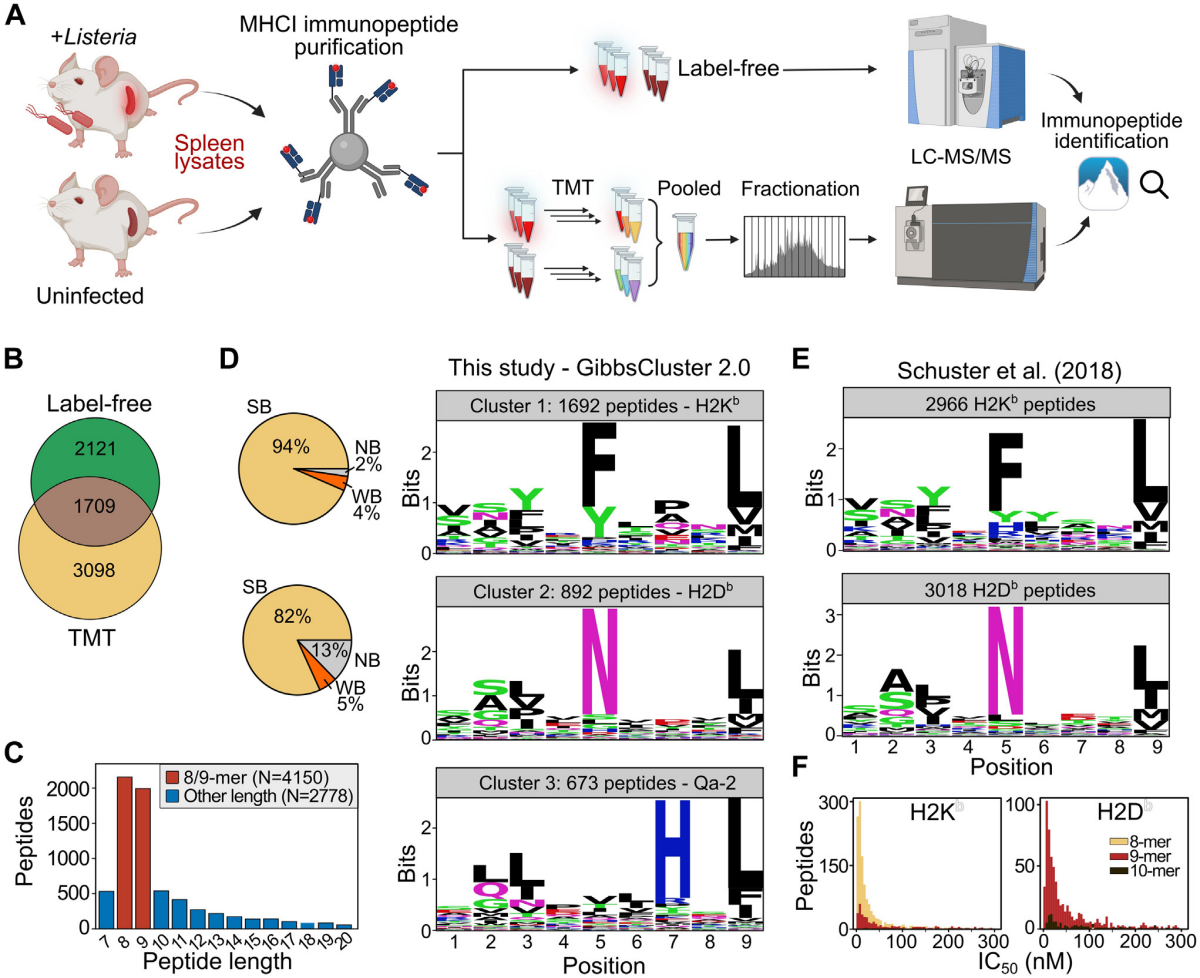
**Identification of mouse MHC class I immunopeptides after *Listeria* infection.***A*, three replicates of *Listeria*-infected and uninfected mouse spleens were homogenized using a mild lysis buffer and MHC class I–epitope complexes were purified as described (26, 86). One-half of the resulting immunopeptides were analyzed by label-free LC-MS/MS analysis on a Q Exactive HF mass spectrometer, while the other half was differentially labeled by TMTpro, pooled, and fractionated into 12 fractions prior to LC-MS/MS analysis on a Fusion Lumos. Database searching was carried out using PEAKS Studio 11 (29). Created with BioRender.com. *B*, Venn diagram showing the number of overlapping immunopeptides identified in the label-free and TMT workflow. *C*, peptide length distribution representing the typical dominance of 8- and 9-mers (*red* bars) in the mouse MHC class I–presented immunopeptidome. *D*, GibbsCluster2.0 clusters 1 to 3 consisting of H2K^b^, H2D^b^, and Qa-2 associated peptides, respectively. Sequence logos were constructed based on the GibbsCluster2.0 alignment cores of peptides in each cluster. Pie charts displaying NetMHCpan-4.1b binding level predictions to H2K^b^ and H2D^b^ alleles for cluster 1 and 2, respectively. Qa-2 is not an available allele for NetMHCpan-4.1b prediction. *E*, sequence logos of unique, high-confidence H2K^b^ (*top*), and H2D^b^ (*bottom*) peptides identified by Schuster *et al*. (45). Given the different peptide lengths, sequence logos were constructed using GibbsCluster2.0 alignment cores (single cluster per allele). *F*, histogram of NetMHCpan-4.1b predicted MHC-binding affinity (IC_50_) below 300 nM for H2K^b^ (*top*) and H2D^b^ (*bottom*) MHC alleles. Bars were colored according to peptide length (8-, 9-, and 10-mer).

To identify novel *Listeria* antigens presented in mice, we isolated MHC I–presented peptides from the spleens of infected C57BL/6 mice or saline-injected controls following 72 h of infection. We inoculated the mice using intravenous infection with 4.1 × 10^6^ CFUs of *L. monocytogenes* EGD per mouse. Spleens from six to seven different animals were pooled to generate three infected and three uninfected biological replicate samples. Purified immunopeptides from each sample were analyzed by LC-MS/MS, both in a label-free approach and after TMT labeling (Fig. 1*A*). In the latter case, peptides were tagged by distinct isobaric labels, pooled, and prefractionated prior to LC-MS/MS analysis in order to maximize the detected immunopeptide repertoire (26, 44).

Database searching with PEAKS Studio 11 identified a total of 6928 peptides including 6891 mouse self-peptides and 37 *Listeria* peptides at a spectral FDR below 1% (Data S1). Only 1709 (24.7%) of the identified peptides were detected by both label-free and TMT analysis, indicating the complementarity of both approaches (Fig. 1*B*). As previously reported (44), TMT labeling resulted in a higher proportion of multiply charged peptide precursors and detectible b-ions than unlabeled peptides (Supplemental Fig. S1). In line with MHC class I–binding motifs and previous mouse immunopeptidomics studies (45), 4150 (59.9%) of the identified peptides were 8- or 9-mer peptides (Fig. 1*C*). Unbiased alignment and clustering of 8- and 9-mer peptides using GibbsCluster2.0 (33) revealed four clusters (Supplemental Fig. S2). Cluster 1 (1692 peptides) and cluster 2 (892 peptides) corresponded to H2K^b^- and H2D^b^-associated peptides, respectively, evidenced by their strong predicted binding affinity (netMHCpan-4.1 %Rank <0.5) to H2K^b^ (94% of cluster 1) and H2D^b^ (82% of cluster 2) class I MHC molecules expressed by C57BL/6 mice (Fig. 1*D* and Data S1). In addition, these sequence motifs matched those derived from the draft map of the murine MHC class I immunopeptidome (Fig. 1*E*) (45). Moreover, 1807 of the H2K^b^/H2D^b^ strong binders in our study (63%) overlapped with this draft map while 1593 peptides (55%) overlapped with the spleen-specific map (Data S1). In addition, peptides associated with H2K^b^ were predominantly 8-mers (76% strong binders), while H2D^b^-associated peptides were mainly 9-mers (79% strong binders) (Fig. 1*F*) (Data S1*A*). Next to the anticipated sequence motifs of these classical MHC class Ia alleles, 673 peptides constituting cluster 3 revealed an additional sequence motif with two C-terminal anchor residues: His at P7 and Leu at P9 (Fig. 1*D*). This motif is in line with reported peptide-binding preferences of the nonclassical MHC class Ib Qa-2 allele (46, 47, 48, 49), which is known to exert cross-reactivity with the employed M1/42 antibody (50). Together, we identified more than 6000 MHC class I immunopeptides presented in *Listeria*-infected mice. The peptide length distribution, predicted binding affinity, and clustering into expected MHC binding motifs supported *bona fide* detection of the immunopeptides and high quality of the dataset.

### Detecting High Confident *Listeria* Immunopeptides

**Figure 2:**
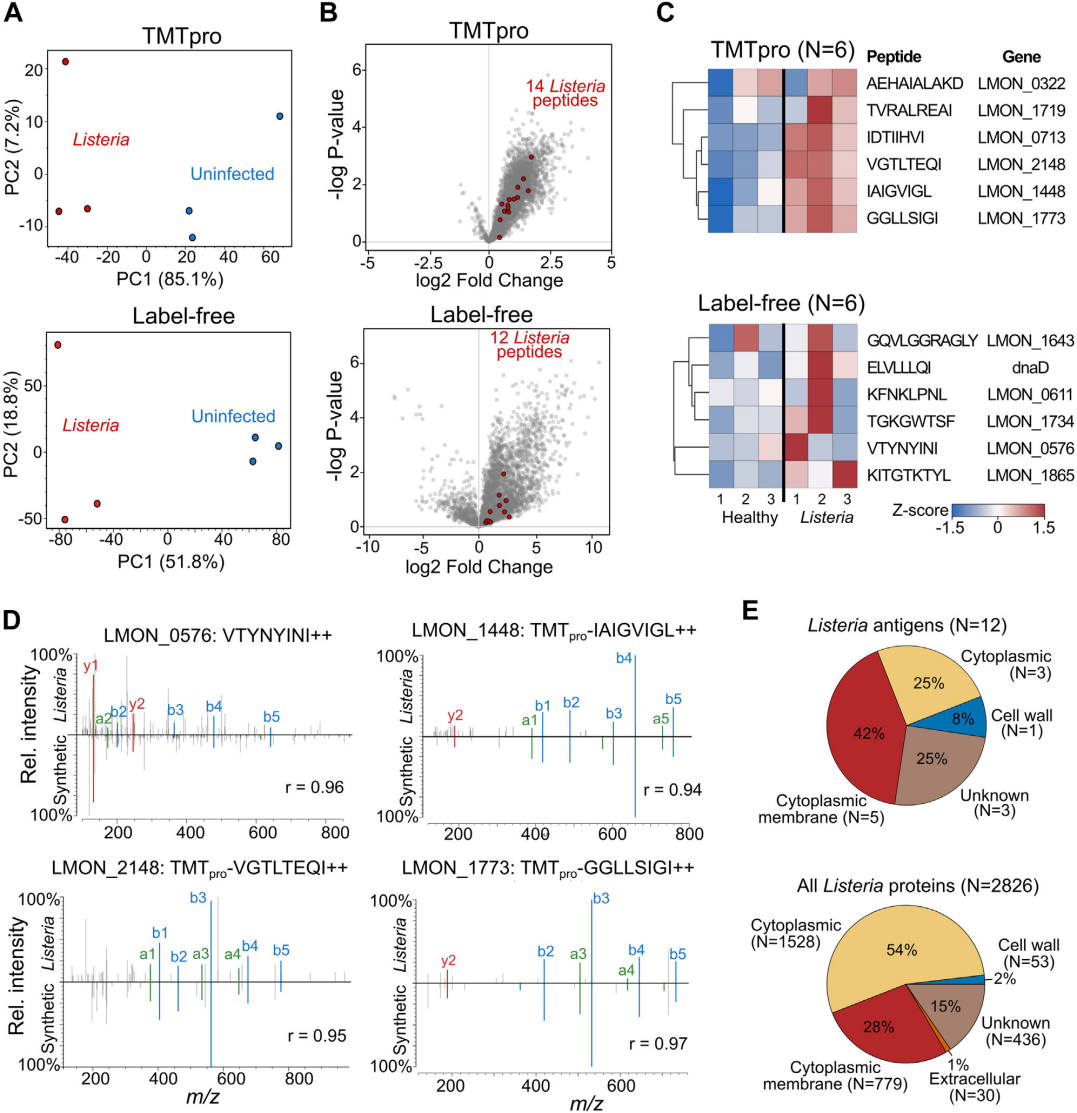
**Detection of high confident *Listeria* immunopeptides.***A*, principal component analysis (PCA) of peptide intensities coherently cluster the *Listeria*-infected (*red*) and uninfected samples (*blue*). *B*, volcano plots indicating the higher abundance of the *Listeria* peptides (*red*) in infected samples. *C*, heatmap of z-scored *Listeria* peptide intensities in the TMT and label-free samples. *D*, all 12 high-confident *Listeria* immunopeptide sequences were synthesized to compare their synthetic and experimental fragmentation spectra, confirming the *bona fide* identification of the four peptides shown here and in Fig. S3. The correlation coefficient r is shown for each *Listeria*-synthetic peptide pair (see methods). *E*, subcellular localization prediction of the 12 identified *Listeria* antigens (*top*) and the entire *Listeria* proteome (*bottom*) by PSORTdb 4.0 (52).

With their detection challenged by their low abundance, the 37 *Listeria* peptides represented only ∼0.5% of the identified 6928 peptides. Adopting the principle of a 1% FDR threshold, 69 false positives can be anticipated, warranting stringent selection of high-confident *Listeria* peptides. To this end, we compared immunopeptide abundances between the infected samples and the uninfected controls. For both label-free and TMT-labeled datasets, infected and uninfected samples clustered coherently (Fig. 2*A*) and 26 *Listeria* peptides could be discerned with an average higher abundance in the infected samples, as expected (Fig. 2, *B* and *C*). Half of these *Listeria* peptides (13/26) were 7-mers, which is incompatible with MHC class I peptide binding (51). After removing these 7-mers, we synthesized the remaining 13 *Listeria* peptides to compare their experimental fragmentation spectrum with the spectrum of their synthetic counterpart. The synthetic and experimental spectra for all peptides except ISGSRLSI showed a high overlap with a Pearson correlation coefficient ranging between 0.67 and 0.99, confirming correct bacterial immunopeptide identification (Figs. 2*D* and Supplemental Fig. S3). Given that ISGSRLSI likely is a wrong spectrum-to-peptide assignment, we finally retained 12 high confidence *Listeria* peptides. In line with anticipated mouse MHC binding, 11 out of 12 of these high confidence peptides were of length 8 or 9 (Fig. 2*C*). Aside from the *Listeria* peptides, many mouse self-peptides were strongly upregulated in the infected samples. For instance, 447 peptides stemming from 361 mouse proteins showed a significant increase in both label-free and TMT samples (adj. *p* value ≤ 0.05 and fold change >2). Functional analysis revealed that these proteins were often involved in antigen presentation and immune-related processes, as expected (Supplemental Fig. S4).

Interestingly, none of the high confidence *Listeria* epitopes were documented in the IEDB nor were they detected in our previous study on infected human cell lines (26). We did however retrieve a 9-mer peptide from the internalin-like protein LMON_0611, a bacterial protein from which another peptide was also presented on infected human HeLa cells (26). Similarly, we found 9-mers from the DNA translocase FtsK and the Flagellar biosynthesis protein FliS, which are already listed with other epitopes as *Listeria* antigens in IEDB. Of the 12 *Listeria* proteins identified, subcellular prediction with PSORTdb 4.0 (52) assigned five proteins (42%) as associated with the cytoplasmic membrane, one protein (LMON_0611) to the cell wall, three proteins as cytoplasmic, and three proteins with unknown subcellular location (Fig. 2*E*). Given that 28% of all *Listeria* proteins are predicted as membrane-associated (Fig. 2*E*), we thus observed a relative enrichment of such peripheral proteins that are likely more accessible and presented to the host machinery (24, 26, 53, 54, 55, 56). Taken together, from the spleens of infected mice, we detected 12 MHC class I–presented peptides from 12 *Listeria* proteins, including previously described antigens and proteins present at the bacterial periphery.

### Screening for Novel *Listeria* CD8^+^ T Cell Epitopes

**Figure 3:**
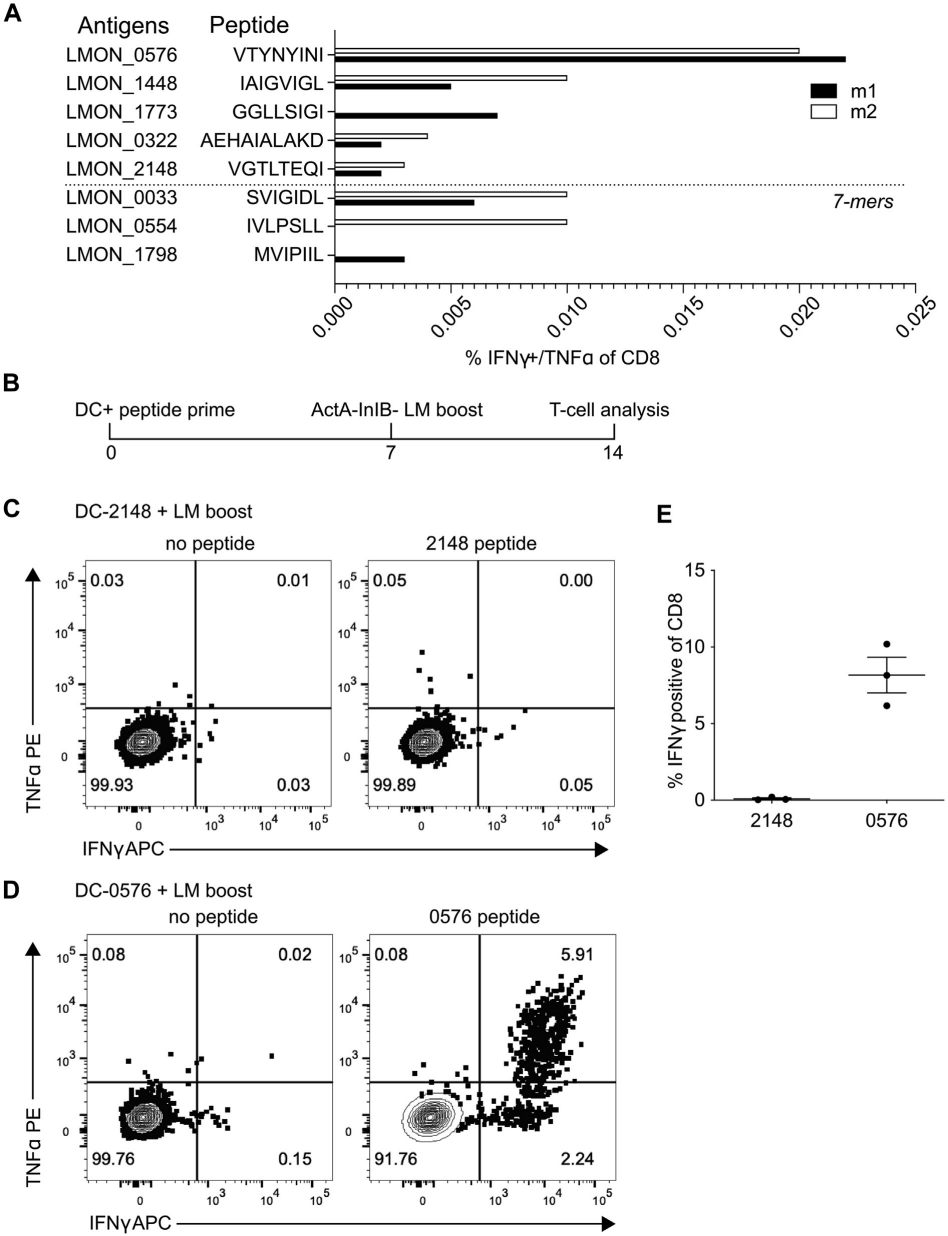
**CD8 T cell responses of MHC class I–associated peptides after DC + peptide prime and Δ*actA-*Δ*inlB-L. monocytogenes* booster immunization.***A*, C57Bl/6 mice were infected i.v. with Δ*actA-*Δ*inlB-L. monocytogenes*. Seven days later, splenocytes were harvested and stimulated with the indicated peptides (1 μM final concentration) for 5 h in the presence of brefeldin A prior to the detection of CD8 T cell–derived IFN-ɣ and TNF. Data presented are from two mice (m1 and m2) from one representative experiment of two with similar results. N = 4 mice total analyzed for all peptides. *B*, experimental design. C57Bl/6 mice were immunized i.v. with dendritic cells (DC) coated with the identified peptides derived from LMON_0576 or LMON_2148 antigens. Seven days later, immunized mice were boosted with Δ*actA-*Δ*inlB-L. monocytogenes* (LM). Fourteen days after boosting, splenocytes were harvested and stimulated with the indicated peptides (1 μM final concentration) for 5 h in the presence of brefeldin A prior to the detection of CD8 T cell–derived IFN-ɣ and TNF. *C*, representative flow plots of IFN-ɣ and TNF in gated CD8 T cells from DC-2148 peptide primed, LM boosted mice. *Left* panel, no peptide stimulation; *right* panel, LMON_2148 peptide stimulation. *D*, representative flow plots of IFN-ɣ and TNF in gated CD8 T cells from DC-0576 peptide primed, LM boosted mice. *Left* panel, no peptide stimulation; *right* panel, LMON_0576 peptide stimulation. Numbers represent % of cells in each quadrant. *E* cumulative data from three mice in each group in one experiment that is representative of two similar experiments.

We next asked the question whether our newly identified *Listeria* immunopeptides are capable of eliciting specific T cell responses. Indeed, *bona fide* pathogen-derived CD8^+^ T cell epitopes are defined not only by binding to MHC class I but also through their ability to react with or stimulate responses from subpopulations of T cells in infected mice (57). To address the latter issue, mice were immunized with a sublethal dose of Δ*actA*Δ*inlB*-*L. monocytogenes* derived from strain 10403s (58). Seven days later, splenocytes from immune mice were stimulated *in vitro* by five selected candidate epitopes at a final concentration of 1 μM for 5 h in brefeldin A followed by fixation and detection of surface CD8 and intracellular IFN-ɣ and TNF expression as a functional readout of antigen-specificity. Simultaneous detection of both cytokines was used to minimize the impact of background cytokine detection. The candidate epitopes were selected based on their predicted strong MHC binding (VTYNYINI and IAIGVIGL) (Data S2) or very high synthetic spectral correlation (GGLLSIGI, AEHAIALAKD, and VGTLTEQI) (Figs. 2*D* and Supplemental Fig. S3). In addition, three 7-mer peptides (SVIGIDL, IVLPSLL, MVIPIIL) that were removed as high confident *Listeria* immunopeptides were included as negative control. The VTYNYINI peptide derived from LMON_0576, predicted to be a strong binding H2Kb epitope (Data S2), stimulated the highest fraction of IFN-ɣ^+^TNF^+^ CD8^+^ T cells from both immunized mice in this initial screen, suggesting that it may serve as a *bona fide* new epitope from *Listeria*, while the other candidate epitopes stimulated to background levels similar to the negative controls (Fig. 3*A*).

Although consistent, the magnitude of the CD8^+^ T cell response against VTYNYINI from LMON_0576 was modest in mice receiving a single priming immunization with *L. monocytogenes*. To further assess the veracity of our initial screening, we turned to an approach (40) to use peptide-loaded DCs followed by boosting with Δ*actA*Δ*inlB- L. monocytogenes* to amplify peptide-specific CD8^+^ T cell responses (Fig. 3*B*). At 7 days post boost, splenocytes from DC-2148 (Fig. 3*C*) or DC-0576 (Fig. 3*D*) primed mice were left unstimulated or stimulated with the 1 μM cognate peptide for the detection of intracellular IFN-ɣ and TNF. DC-2148 primed, *Listeria*-boosted mice made no detectable CD8^+^ T cell response to the LMON_2148 peptide compared to unstimulated control splenocytes (Fig. 3, *C* and *E*). In contrast, DC-0576 primed, *Listeria*-boosted mice made robust IFN-ɣ^+^TNF^+^ CD8^+^ T cell responses to the LMON_0576 peptide, representing 5 to 10% of the CD8 T cell compartment (Fig. 3, *D* and *E*). As additional negative controls, two 7-mer peptides (derived from LMON_0033 and LMON_0554) were tested in a similar manner (data not shown), but only the peptide from LMON_0576 stimulated detectable CD8^+^ T cell responses. These data provide additional evidence that the LMON_0576-derived peptide is a *bona fide* CD8^+^ T cell epitope from *Listeria*.

### Biological Potency of the LMON_0576-Derived Peptide

**Figure 4:**
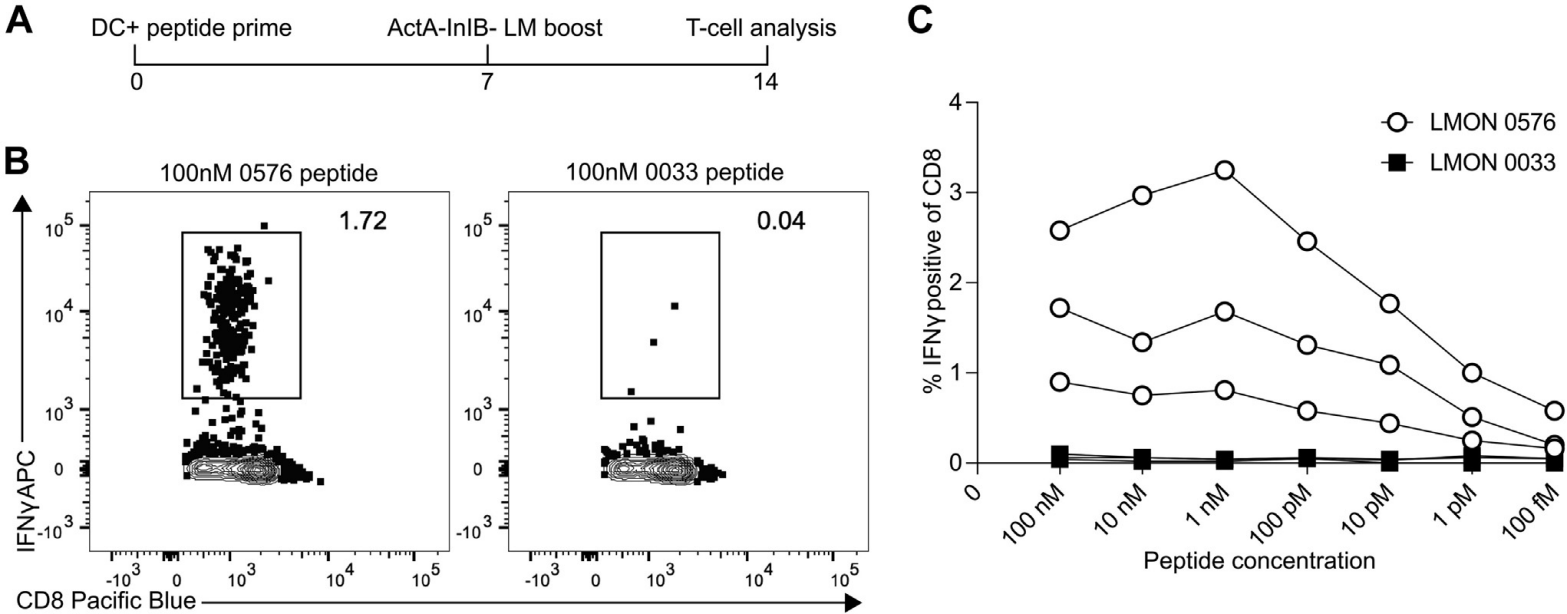
**Dose titration of selected peptides.***A*, experimental design, same as in Fig. 3 except that the peptide derived from LMON_0576 was compared to a 7-mer peptide derived from LMON_0033 as negative control. *B*, representative IFN-ɣ production from gated CD8 T cells at saturating 100 nM peptide dose. *C*, dose titration curves of IFN-ɣ production from individual mice.

Peptides representing natural CD8^+^ T cell epitopes are extremely potent stimulators of cytokine production from antigen-specific CD8^+^ T cells, with biological activity in the picomolar-femtomolar range in *in vitro* assays (40). To evaluate the potency of the LMON_0576-derived peptide, we immunized groups of mice with DC-0576 or DC-0033 peptide and boosted with Δ*actA*Δ*inlB-L. monocytogenes* (Fig. 4*A*). Seven days post boost, splenocytes from individual mice were stimulated with titrating amounts of cognate peptide, from 100 nM to 100 fM final concentration. All mice that received DC-0576 prime, *Listeria* boost made IFN-ɣ^+^TNF^+^ responses in response to the cognate peptide ranging from 1 to 3% of CD8^+^ T cells (Fig. 4, *B* and *C*). IFN-ɣ^+^TNF^+^ CD8^+^ T cells were not detected at any peptide concentration in mice primed and stimulated with LMON_0033-derived peptide (Fig. 4*C*). Consistent with the potency of previously identified *bona fide* MHC class I–restricted epitopes (59), IFN-ɣ^+^TNF^+^ CD8^+^ T cells were still detected above background at LMON_0576-derived peptide concentrations below 10 pM (Fig. 4*C*). Together, the robust CD8^+^ T cell responses induced by DC-0576 prime, *Listeria* boost and the biological potency of the LMON-0576-derived peptide in stimulating IFN-ɣ^+^TNF^+^ responses from CD8 T cells strongly support its designation as a new MHC class I–restricted epitope from *Listeria*.

### Protection by LMON_0576 Immunization

**Figure 5:**
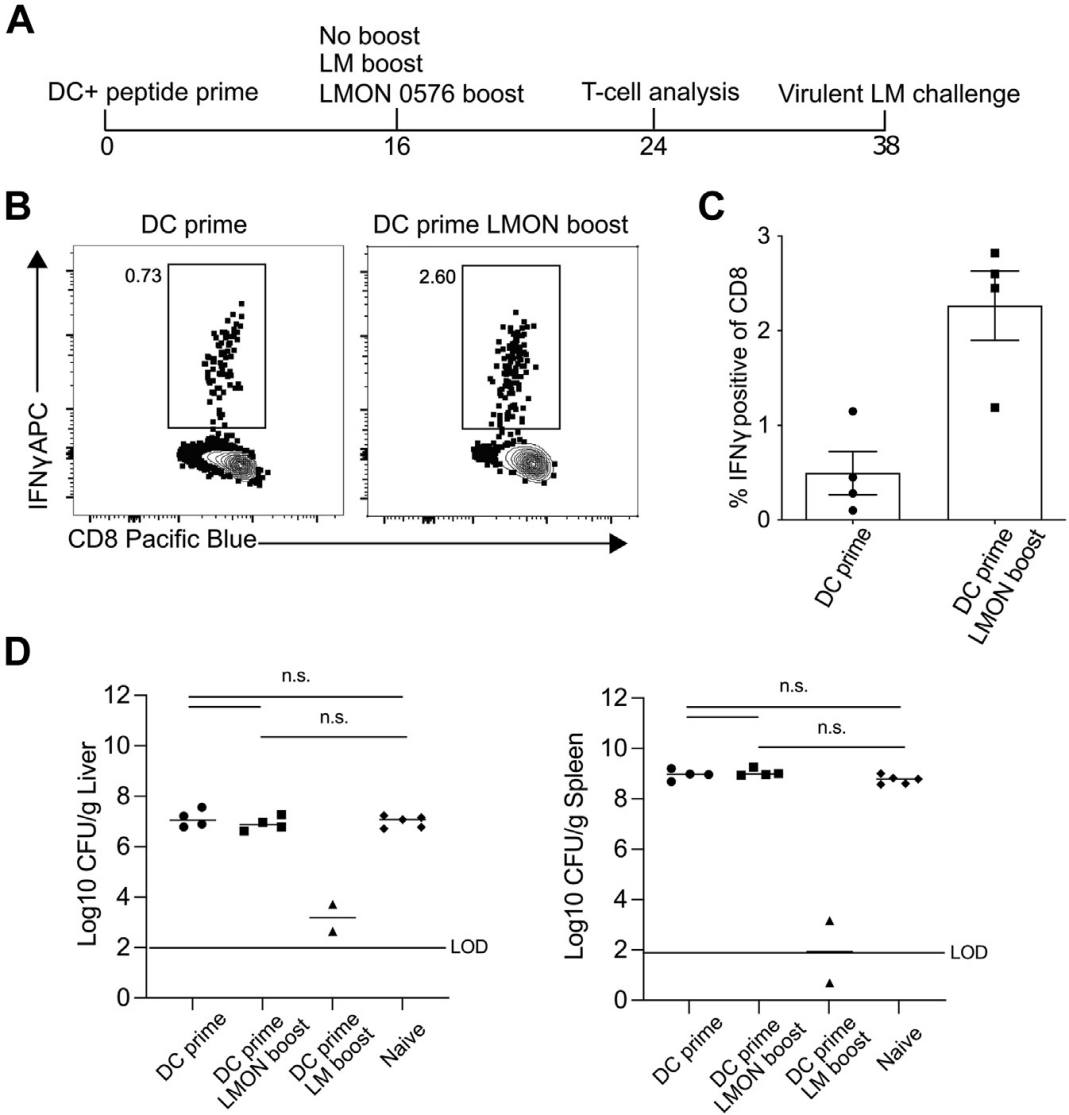
**Assessment of protective immunity after DC-0576 priming and boosting with mRNA-LNP vaccine expressing the LMON_0576 antigen.***A*, experimental design. A group of C57Bl/6 mice were DC + 0576 peptide primed. Sixteen days later, some of these mice were boosted with an mRNA vaccine expressing the LMON_0576 antigen or Δ*actA-*Δ*inlB-L. monocytogenes*. *B*, representative flow plots of LMON_0576 peptide-stimulated IFN-ɣ production by gated CD8 T cells from the blood in the indicated group at 24 days after initial DC + 0576 prime. *C*, cumulative data showing % IFN-ɣ production from five mice per group at 24 days after DC + 0576 prime. *D*, immunized mice and naïve controls were challenged i.v. with virulent LM. Colony-forming units per gram of liver (*left*) and spleen (*right*) at 3 days post challenge. Each dot is an individual mouse. Data analyzed by one-way ANOVA.

While CD8^+^ T cell responses detected after viral infection are generally protective, several studies from us and others show that this is not always the case for CD8^+^ T cell responses detected against more complex pathogens such as intracellular bacteria, including *Listeria* (53), or parasites such as *Plasmodium berghei* (60) or *Toxoplasma gondii* (61). To assess this, we primed three groups of mice with DC-0576 peptide (Fig. 5*A*). One group remained unboosted (DC prime) and one group received Δ*actA-*Δ*inlB-L. monocytogenes* boosting (DC prime LM boost) 16 days after prime. The latter group served as a positive control because it contained CD8 T cell responses against all *Listeria* epitopes. To address immunity against the LMON_0576-derived epitope, the third group of DC-0576 primed mice was boosted with an LNP vaccine containing 2 μg of a codon-optimized mRNA encoding the LMON_0576 antigen (DC prime LMON boost). LMON_0576-derived peptide-specific CD8^+^ T cell responses in the DC prime and DC prime LMON boost groups were determined 8 days after boosting (Fig. 5, *B* and *C*) and were elevated in the DC prime LMON boost group. Immunized mice and age-matched naïve mice were then challenged with a lethal dose of virulent *Listeria* at day 38 after initial immunization and bacterial numbers in the spleen and liver were determined 3 days later (Fig. 5*D*). Challenged naïve mice had high bacterial load in both the spleen and liver, whereas, as expected, mice that received *Listeria* boosting contained low numbers of bacteria in both organs. In contrast, mice of the DC prime or DC prime LMON boost group exhibited no control of infection, in either the spleen or liver. Thus, while we provide strong evidence that the LMON_0576-derived peptide is a *bona fide* MHC class I–restricted epitope from *Listeria*, CD8^+^ T cells specific for this epitope were not capable of providing measurable control of challenge infection.

## Discussion

*L. monocytogenes* is an intracellular model bacterium, widely used to study CD8^+^ T cell responses in immune and vaccine studies (19, 21, 22). However, the read-out of these responses in murine models has so far been limited to a handful of bacterial epitopes known to be presented on MHC class I molecules (27, 62). To identify novel *Listeria* epitopes, we screened for bacterial immunopeptides presented in the spleen of infected C57BL/6 mice by immunopeptidomics, an approach based on pull down of MHC class I molecules from the homogenized organs followed by elution and MS-based identification of the bound immunopeptides. Immunopeptidomics workflows have improved substantially in recent years, and we recently employed this technology to investigate the MHC class I immunopeptidome of *Listeria*, reporting 68 bacterial immunopeptides presented on infected human cells (26). In the present study, we detected 12 MHC class I peptides presented on murine cells infected with *L. monocytogenes* EGD. To the best of our knowledge, none of these peptides were previously reported, while on the antigen level, the internalin-like protein LMON_0611 was identified before in our study on human cells with another epitope (26). The *Listeria* immunopeptides were identified among more than 6000 mouse self-peptides with expected length distribution, binding affinity, and clustering into the H2D^b^, H2K^b^, and Qa-2 MHC-binding motifs. In the infected samples, we observed a strong upregulation of self-peptides derived from proteins involved in immune-related processes. This likely explains the rather modest overlap with the draft map of the murine MHC class I immunopeptidome that was compiled from tissues of untreated healthy mice (45). In addition, unlike the latter study that employed the H2D^b^- and H2K^b^-specific antibodies B22-249.R1 and Y-3, respectively, we used the M1/42 antibody that exhibits a broad H2 MHC class I specificity (63), including reactivity with MHC class Ib Qa-2 allele (50). In line with this, Gibbs clustering revealed a cluster of 673 peptides (out of 4150 or 16% 8- or 9-mers) containing two Qa-2 dominant C-terminal anchor residues (His at P7 and Leu at P9, together with Phe and Ile) corresponding to the reported specificity for the Qa-2 allele (46, 47, 48, 49). Interestingly, we also identified the Qa-1b leader peptide AMAPRTLLL (or Qa-1 determinant modifier, Qdm) in our dataset (Data S1), suggesting that M1/42 also pulls down this allele next to Qa-2. Our pull down results thereby recapitulate immunopeptidomics data obtained by an earlier M1/42 pulldown (50) as well as a study performing mild acidic elution from a mouse cell line, which also revealed a significant fraction of Qa-2 immunopeptides next to H2D^b^ and H2K^b^ binders (64). This indicates that the M1/42 antibody might be preferred over allele-specific antibodies for untargeted epitope detection, especially in the context of antigen discovery for vaccine development.

Similar to our previous study (26), we employed a hybrid immunopeptidomics approach combining label-free and TMT-labeling. The limited overlap between unlabeled and TMT-labeled peptides likely resulted from altered peptide ion characteristics, corroborating previous findings (44). Especially an increased charge state and b-ion coverage was apparent for TMT-labeled immunopeptides (Supplemental Fig. S1), features that also helped to inspect and correctly assign synthetic spectra from *Listeria*-derived immunopeptides. Indeed, a particular challenge in immunopeptidomics studies for xeno-epitope discovery is the correct identification of only a few pathogen peptides in an ocean of host peptides. Here, it is routine practice to combine host and pathogen proteome for database searching and FDR assessment. Preferably, class-specific FDR scoring for pathogen and host separately would be performed, though this often proves overly strict and difficult by the sheer low abundance and presentation of bacterial epitopes. Instead, we resorted to a 1% FDR threshold, a more stringent cutoff compared to other bacterial immunopeptidomics studies (24, 25). Moreover, spectral similarity and retention time deviations to deep learning–predicted features were leveraged since the ‘Deep learning boost’ embedded within PEAKS Studio was enabled. Together, our hybrid labeling approach in combination with powerful search and stringent FDR settings led to a high confident set of six unlabeled and six TMT-labeled *Listeria*-derived immunopeptides for which fragmentation spectra were confirmed by synthetic peptides.

When we tested five *Listeria* immunopeptides for their capacity to elicit specific CD8^+^ T cell responses, we could only confirm the LMON_0576 peptide VTYNYINI as a *bona fide* T cell epitope. It has been recognized for some time that not all peptides isolated from MHC class I molecules are recognized by CD8^+^ T cells. Even in infected cells, many MHC I–bound peptides will derive from self-proteins, where the host has used central and peripheral mechanisms to limit host-responsive CD8 T cell responses (65). Additionally, despite the potentially enormous number of unique TCRs expressed within a single host (66), CD8^+^ T cells that recognize a particular peptide–MHC complex may be rare or nonexistent due to constraints of MHC-biased positive and negative selection in the thymus (67). As a consequence, it is perhaps not surprising that only one of the 12 candidate MHC I–bound peptides identified from *L. monocyogenes*–infected spleens could be clearly identified as a *bona fide* novel CD8^+^ T cell epitope.

We initially screened individual candidate peptides for their ability to induce cytokine (IFN-ɣ and TNF) production from *L. monocytogenes*–induced CD8^+^ T cells as a measure of functional relevance. Concurrent expression of these cytokines was used as a cutoff to minimize the confounding effects of background production of single cytokines due to the low magnitude responses observed. This approach permitted us to identify the VTYNYINI peptide derived from the LMON_0576 antigen that reproducibly stimulated IFN-ɣ^+^TNF^+^ CD8^+^ T cells above the background established with three 7-mer peptides. However, the frequencies of these specific CD8 T cells were only modestly elevated in mice receiving a single immunization with Δ*actA-*Δ*inlB-L. monocytogenes*. The reasons for this low response remain to be determined but could result from a low precursor number of naïve CD8^+^ T cells prior to immunization or inefficient processing and presentation of the LMON_0576 antigen. LMON_0576 is an uncharacterized bacterial surface protein with two predicted transmembrane and three MucBP domains (mucin-binding protein, Pfam PF06458) (68, 69). According to the Pfam database, MucBP-containing proteins are primarily present in *Lactobacillales* and *Listeria* bacterial species. MucBP domains are typically arranged as tandem repeats that were shown to trigger adhesion to mucus in *Lactobacillus reuteri* 1063 (70) and an increasing number of other bacteria (71). In *Listeria*, besides LMON_0576 (or lmo0576 in the reference strain *L. monocytogenes* EGDe), MucBP domains are found in LPXTG surface proteins, including lmo1413 in EGDe which was shown to bind to type II mucin as an invasin (72). Whether LMON_0576 can similarly promote bacterial entry remains to be established, but transcriptional upregulation upon intracellular growth hints to a possible role in this direction (42, 73). Interestingly, while LMON_0576 exhibits a median 97% sequence identity to its orthologs in 318 fully sequenced *L. monocytogenes* strains, the VTYNYINI epitope is fully conserved in 38% of the strains, including the 10403S strain that was used in the T cell assays (Supplemental Fig. S5).

The low response detected against the LMON-0576–derived peptide required further validation to support its identification as a novel *L. monocytogenes* antigen. This prompted us to employ a rapid prime-boost amplification strategy for studies of antigen-specific CD8^+^ T cells developed in our laboratory (40). In this approach, we generated mature DC in one group of mice, then isolated these cells, loaded them with LMON_0576–derived or other peptides and primed mice by intravenous injection. DC primed mice were then boosted 1 week later with Δ*actA-*Δ*inlB-L. monocytogenes* to provide the endogenous epitope and relevant inflammatory signals (74). This strategy generated robust IFN-ɣ^+^TNF^+^ CD8^+^ T cell responses against the LMON_0576–derived epitope ranging from 3 to 10% of circulating CD8^+^ T cells at 1 week after boosting. In contrast, DC priming with three other candidate epitopes (all negative following initial screening) followed by *L. monocytogenes* boosting did not result in detectable CD8^+^ T cell responses against the cognate peptides. As an additional validation, we examined the potency of the LMON_0576–derived peptide in stimulating cytokine secretion after *in vitro* stimulation of CD8^+^ T cells from DC-0576 or DC-0033 primed, *L. monocytogenes* boosted mice. Cytokine production above background was detected at final LMON_0576-derived peptide concentrations below 10 pM. This biological potency is consistent with *bona fide* epitopes that have been previously characterized (59, 75). Together, these data support the notion that the LMON_0576-derived peptide is a *bona fide* novel CD8 T cell epitope from *L. monocytogenes*.

A major goal of epitope identification is the potential to use this information in developing subunit vaccines to prevent infections or treat malignancies (76). However, recent studies from us and others have shown that detection of an epitope-specific CD8 T cell response in an infected or immunized host does not ensure that those CD8^+^ T cells can contribute to protective immunity (53, 60, 61). This seems to be a particular concern with complex intracellular bacterial and protozoan pathogens that express a large number of foreign antigens that may be compartmentalized within the pathogen or be expressed at low levels in some but not all infected cells. Thus, selection of vaccine candidates requires an additional layer of characterization in addition to the demonstration of antigen-specific CD8^+^ T cells. To address this, we primed mice with DC-0576–derived peptide and boosted with an mRNA-LNP vaccine containing codon-optimized LMON_0576 mRNA. Prime-boosted mice had CD8^+^ T cell responses exceeding 2% of circulating CD8^+^ T cells; however, these mice were not protected from challenge with virulent *L. monocytogenes*. Thus, we must conclude that, while the LMON_0576–derived peptide is a *bona fide* epitope that induces *L. monocytogenes*–specific CD8^+^ T cells, it is not a candidate for inclusion in a subunit vaccine to induce protective immunity. This is in contrast with our previous study on infected human cell lines where LMON_0149 was revealed as a protective vaccine candidate when encoded in an mRNA-LNP vaccine (43). However, LMON_0149 was represented by up to seven different epitopes presented on two different cell lines, whereas in the present study, we only identified a single epitope for LMON_0576. This suggests that to identify vaccine candidates for complex intracellular pathogens, ideally the results of multiple immunopeptidomics screens should be integrated, combining data from different cell and infection models to prioritize antigens presented by multiple epitopes (23).

Despite the successful discovery of a novel *Listeria* epitope, our study was prone to several limitations. First, we did not identify well-established murine *Listeria* epitopes reported in literature. For the H-2K^d^–restricted epitopes LLO 91-99 (59), p60 217-225 (77), p60 449-457 (78), and p60 476-484 (79) absence was expected since C57BL/6 mice do not express the H-2K^d^ allele. However, we did not detect the H-2K^b^–restricted epitope LLO 296-304 (79) presented in C57BL/6 mice nor did we pick up more recently reported epitopes from GAPDH (80). Several reasons might account for this. It is well established that even modern mass spectrometers do not manage to fragment and sequence every peptide ion that enters the instrument. Besides sensitivity limits, restrictions on the speed by which peptides can be sampled for fragmentation lead to a general undersampling, especially in data-dependent acquisition that was used (81). Moreover, compared to our previous study where a multiplicity of infection of 50 led to high *in vitro* infection levels (26), *in vivo* infections are moderated by the host immune system, leading to a dynamic but more controlled infection process. Even though intravenous injection of *Listeria* in mice could result in up to 100 million CFUs in the spleen 72 h post infection (26, 82) that still corresponds to only 1 bacterium/splenocyte assuming that an average mouse spleen consists of 100 million cells (83), while in reality, most cells remain likely uninfected. Together, such relatively low *in vivo* infection levels combined with noncomprehensive MS sampling could explain why we only identified a dozen of *Listeria* immunopeptides. Second, besides under-detection of certain peptides, immunopeptidomics datasets might also contain low levels of contaminating peptides that may be degradation products or result from nonspecific copurification of MHC class II peptides (84). The risk for degradation products was mitigated by preclearing the lysates with a protein A precolumn, also needed to capture endogenous antibodies before MHC pull down, and by removing 7-mer peptides from our list of high confident *Listeria* peptides. Nevertheless, we cannot exclude a low amount of MHC class II contamination since 187 of the identified >12-mer peptides (2.7% out of 6928 peptides) are predicted binders of the class II I-Ab allele. Despite these limitations, our study revealed VTYNYINI from LMON_0576 as a novel H-2K^b^–restricted epitope and valuable research tool to monitor CD8^+^ T cell responses upon *Listeria* infection in mice.

## Data Availability

The mass spectrometry proteomics data have been deposited to the ProteomeXchange Consortium via the PRIDE (85) partner repository with the dataset identifier PXD047750. A detailed overview of the experiment (RAW MS filenames, replicate, fraction and condition) is available in Data S3.

## Supplemental data

This article contains supplemental data (38).

## Conflict of interest

The authors declare no competing interests.
