# Supplementary material for: Immunopeptidomics Mapping of Listeria monocytogenes T Cell Epitopes in Mice
Source: Mol Cell Proteomics. 2024 Aug 13;23(9):100829. doi: 10.1016/j.mcpro.2024.100829 (PMC11414675; doi:10.1016/j.mcpro.2024.100829)
Supplement: Supplemental Figures S1–S5 [file mmc1.docx]

**Supplementary Figures**

**Title**: Immunopeptidomics identification of *Listeria monocytogenes* T cell epitopes in mice

**Contents**:

- Supplementary Figures S1-S5


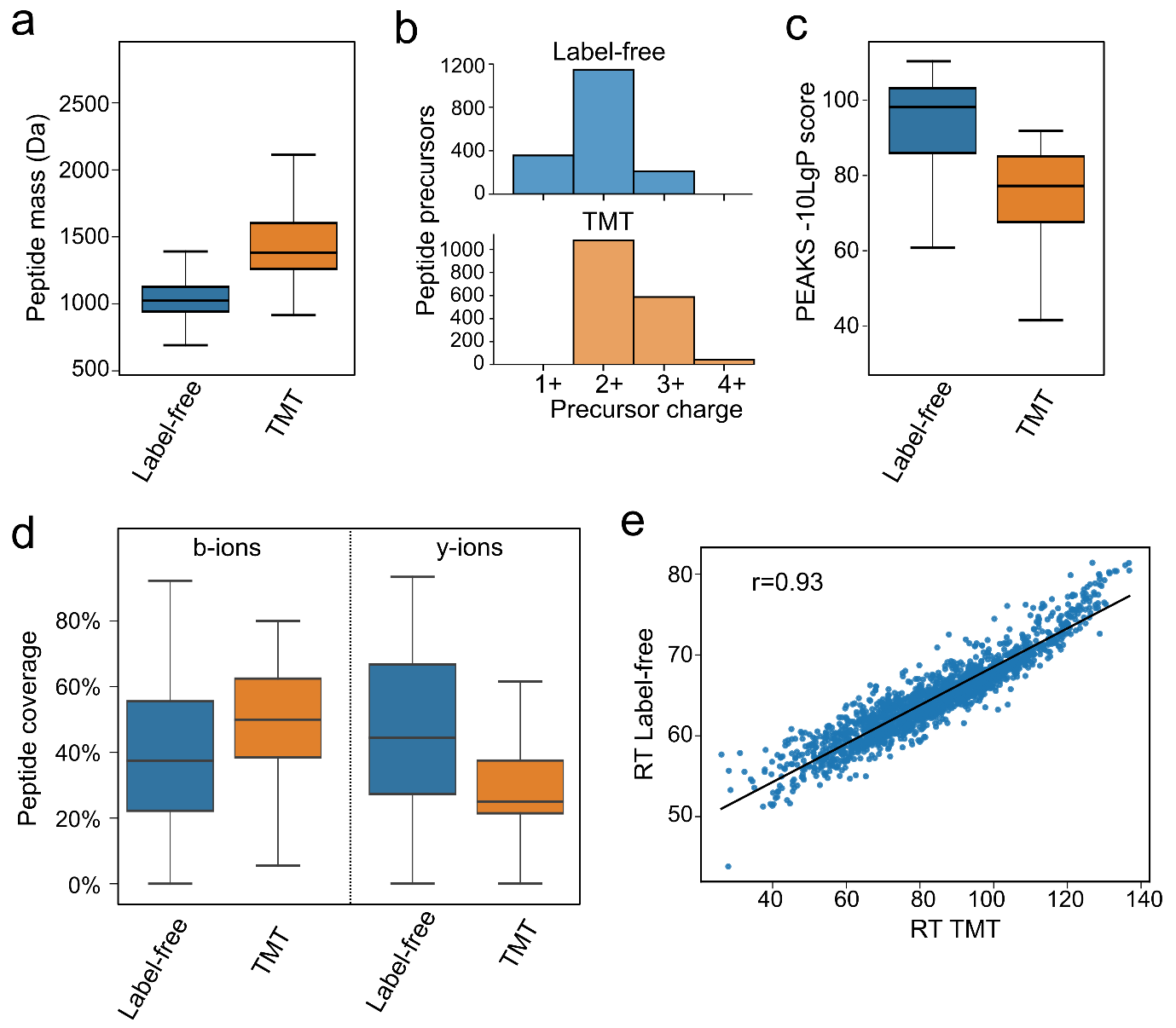


**Figure S1.** **Comparison of identified label-free and TMT-labeled spectral matches** (blue and orange, respectively). Top-scoring (highest PEAKS -10LgP score) peptide-to-spectrum matches for peptide sequences common to label-free and TMT-labeled fractions (Data S1b-c) were compared for multiple properties. (**a**) Box plots showing the distribution of peptide masses in Da. (**b**) Histogram showing the proportion of peptide precursors with a 1+, 2+, 3+ or 4+ charge. (c) Box plots showing the distribution of PEAKS -10LP scores. (**d**) Box plots showing the proportion of peptide residues matched by b-ions (*left*) or y-ions (*right*) (1+ or 2++). The refined peak lists (.MGF) outputted by PEAKS were used to annotate b/y-ions with spectrum.utils (1, 2) at a 0.05 Da tolerance, filtering the top 300 peaks with minimum 2% intensity in the spectrum. (**e**) Scatterplot of retention times (in min) for peptide sequences in TMT-labeled (x-axis) and label-free (y-axis) fractions. The Pearson correlation coefficient is 0.93. Note that different LC-MS/MS set-ups were applied for TMT-labeled and label-free fractions (see Methods). (**a,c-d)** All box plots display the median (central line), the 1^st^ to 3^rd^ quartile (box, Q1-Q3) and whiskers denote 1.5 times the interquartile range (IQR) from Q1 and Q3.


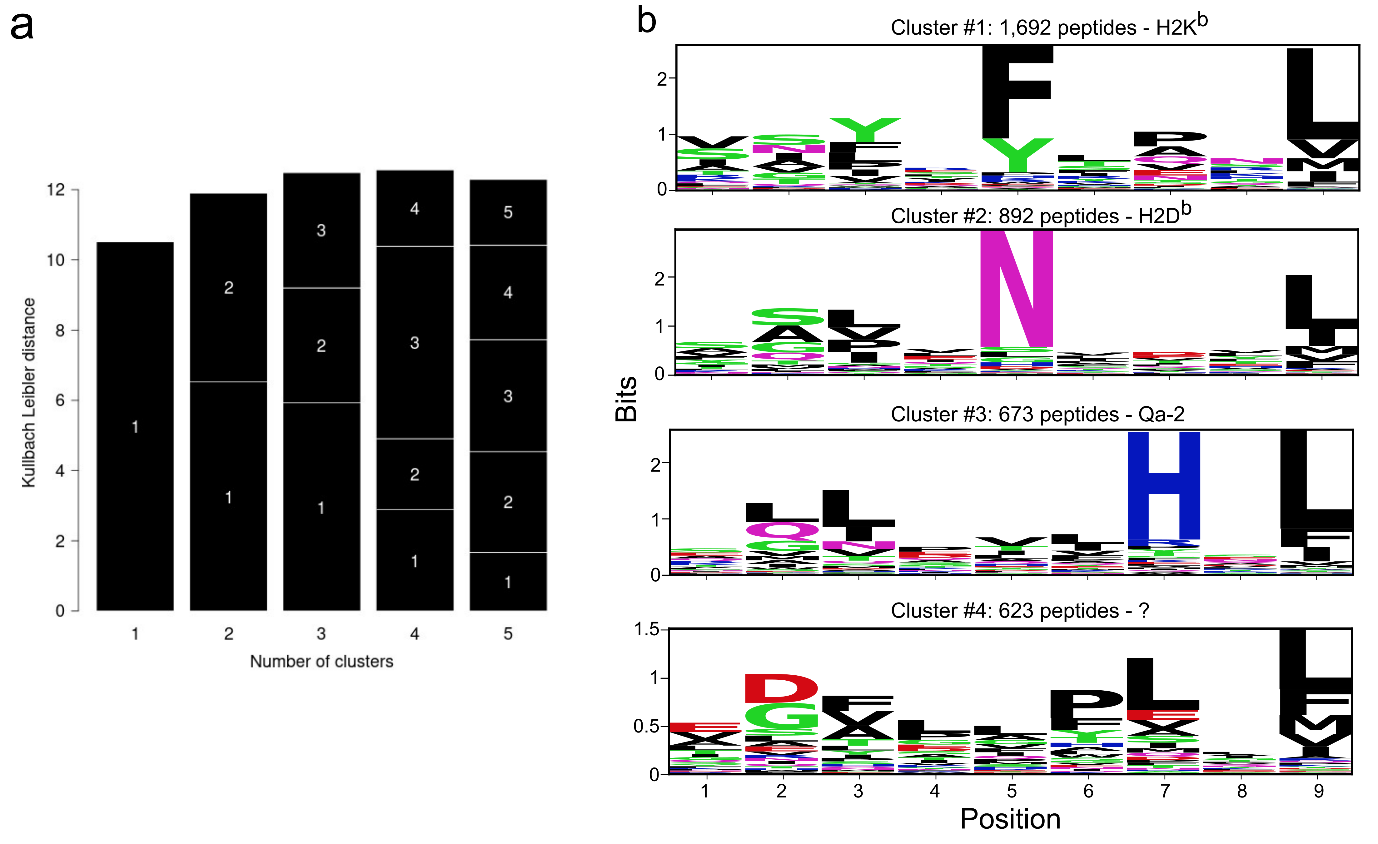


**Figure S2.** **Unbiased alignment and clustering of immunopeptides.** (**a**) GibbsCluster2.0 (5) Kallbach Leibler distance (KLD) suggests four clusters as the optimal solution for the 4,150 identified 8/9-mers. (**b**) Sequence logos for the alignment cores of the peptides per cluster, sorted from largest to smallest cluster (top to bottom). Cluster #1 and #2 matched known H2K^b^ and H2D^b^ motifs (6), while cluster #3 is in line with reported C-terminal dominant anchor residues for Qa-2 (7-10). Cluster 4 is a cluster containing remaining peptides that corresponded less with known H2K^b^, H2D^b^ and Qa-2 sequence motifs. Sequence logos were plotted using Logomaker (4), plotting the GibbsCluster2.0 peptide alignment cores per cluster.


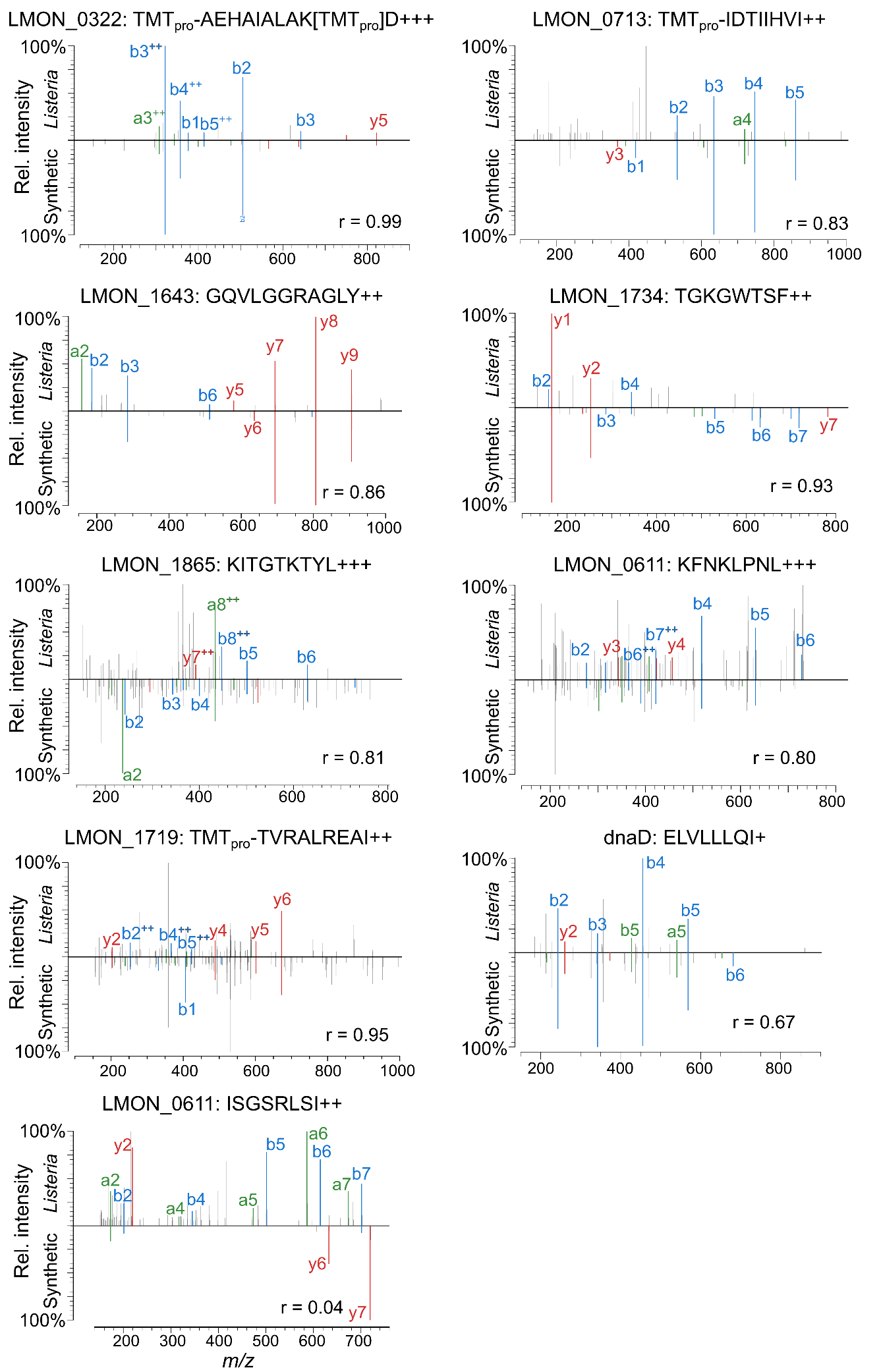


**Figure S3.** **Validation of high confident *Listeria* immunopeptide identification by synthetic peptides**. All ≥ 8-mer *Listeria* immunopeptide sequences were synthesized to compare their synthetic and experimental fragmentation spectra, confirming the bona fide identification for all identified peptides except ISGSRLSI/2+. Synthetic spectra for the other four peptides are shown in Fig. 2d. The correlation coefficient r is shown for each *Listeria*-synthetic peptide pair (see Methods).


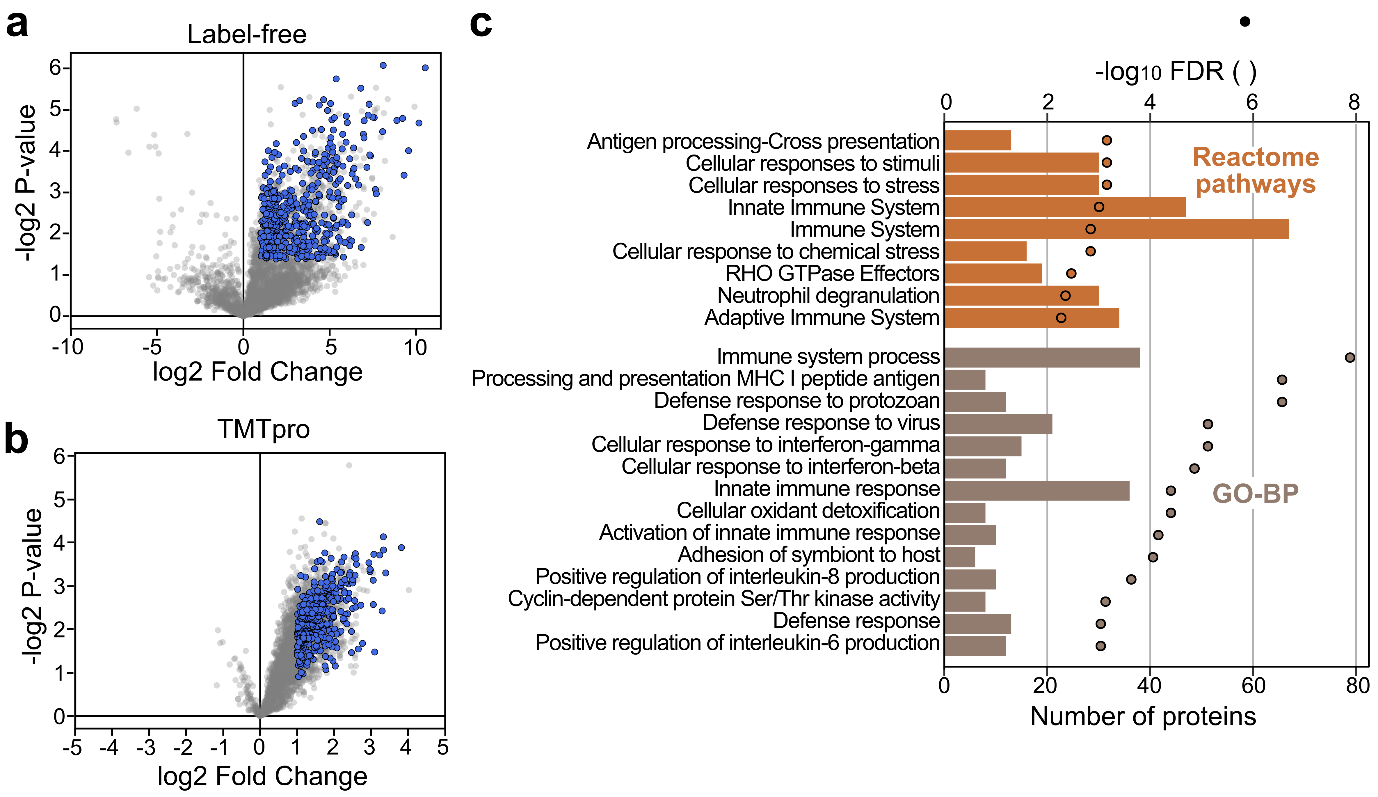
**Figure S4.** **Mouse spleen immunopeptidome changes upon *Listeria* infection.** **(a,b)** Volcano plots indicating the 447 mouse self-peptides (blue) stemming from 361 mouse proteins that are significantly higher abundant (adj. P value ≤ 0.05 and fold change > 2) in infected samples of label-free (a) and TMTpro-labeled (b) fractions. (**c**) Gene set enrichment analysis of 361 mouse proteins with higher abundant presented self-peptides upon *Listeria* infection. The DAVID webserver (11) was used for gene set enrichment, selecting gene ontology – biological process (GO-BP), InterPro protein domains and Reactome pathways as gene sets for testing enrichment. GO-BP and Reactome pathways were displayed if the FDR was below 0.001 and 0.01, respectively.


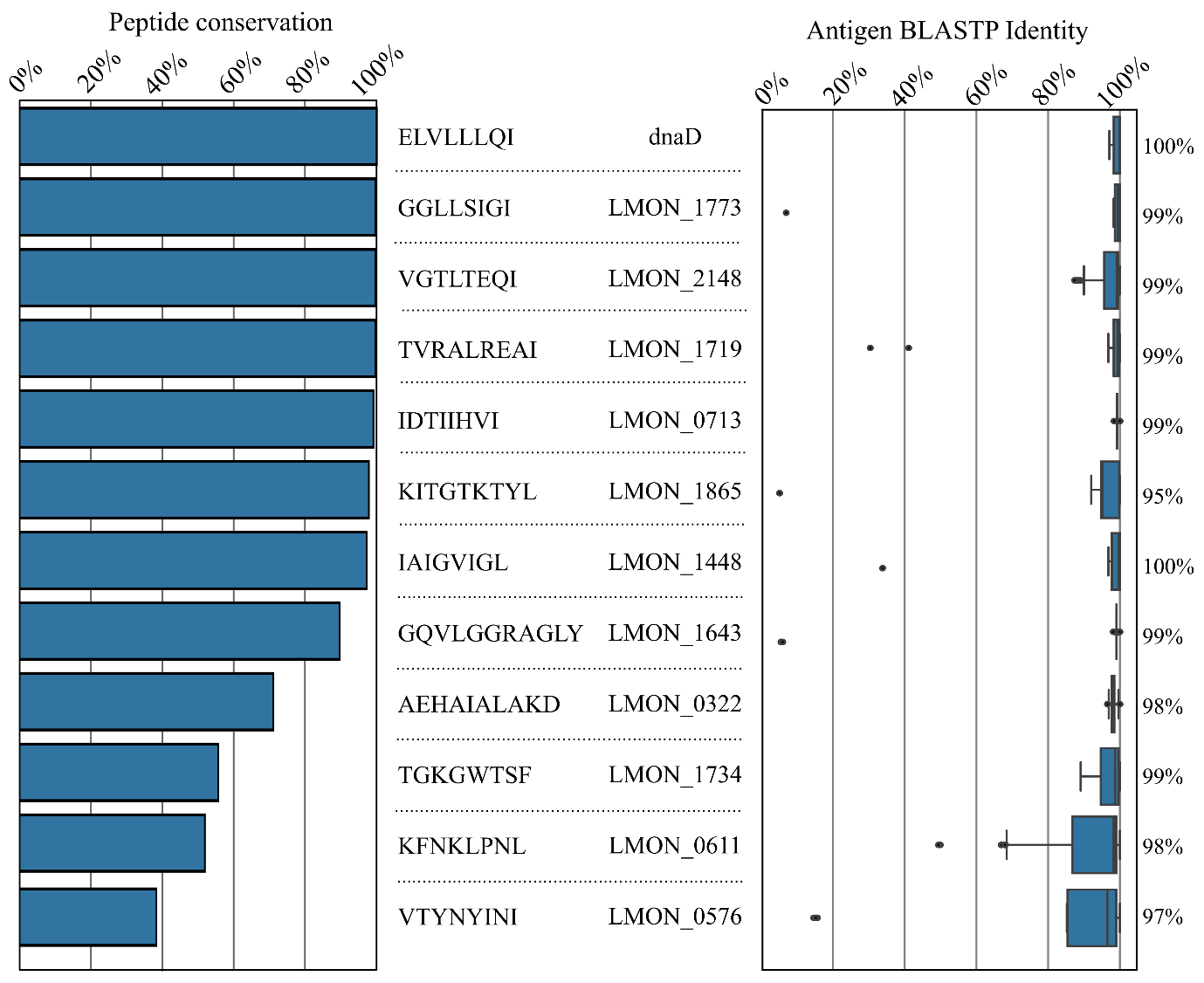


**Figure S5.** **Sequence conservation of identified *Listeria* immunopeptides and antigens.** 318 *Listeria monocytogenes* strains with a complete NCBI RefSeq assembly were used to assess sequence conservation (excluding the EGD strain that was used in this work). Presence of the 12 identified *Listeria* high-confident peptides in each strain was performed by string matching in Python iterating over each proteome FASTA file (*left*). In case of antigen sequence similarity, BLASTP (version 2.9.0+) was used to calculate the number of identical residues (BLASTP ‘nident’) divided by the length of the respective antigen (BLASTP ‘qlen’) the best BLASTP hit per strain (lowest e-value). The distribution of sequence similarity is displayed in a boxplot per antigen, indicating the median % identity (*right*).

**References**

1. Bittremieux, W. (2020) spectrum_utils: A Python Package for Mass Spectrometry Data Processing and Visualization. *Anal Chem* 92, 659-661

2. Bittremieux, W., Levitsky, L., Pilz, M., Sachsenberg, T., Huber, F., Wang, M., and Dorrestein, P. C. (2023) Unified and Standardized Mass Spectrometry Data Processing in Python Using spectrum_utils. *J Proteome Res* 22, 625-631

3. Reynisson, B., Alvarez, B., Paul, S., Peters, B., and Nielsen, M. (2020) NetMHCpan-4.1 and NetMHCIIpan-4.0: improved predictions of MHC antigen presentation by concurrent motif deconvolution and integration of MS MHC eluted ligand data. *Nucleic Acids Res* 48, W449-W454

4. Tareen, A., and Kinney, J. B. (2020) Logomaker: beautiful sequence logos in Python. *Bioinformatics* 36, 2272-2274

5. Andreatta, M., Alvarez, B., and Nielsen, M. (2017) GibbsCluster: unsupervised clustering and alignment of peptide sequences. *Nucleic Acids Res* 45, W458-W463

6. Schuster, H., Shao, W., Weiss, T., Pedrioli, P. G. A., Roth, P., Weller, M., Campbell, D. S., Deutsch, E. W., Moritz, R. L., Planz, O., Rammensee, H. G., Aebersold, R., and Caron, E. (2018) A tissue-based draft map of the murine MHC class I immunopeptidome. *Sci Data* 5, 180157

7. Rotzschke, O., Falk, K., Stevanovic, S., Grahovac, B., Soloski, M. J., Jung, G., and Rammensee, H. G. (1993) Qa-2 molecules are peptide receptors of higher stringency than ordinary class I molecules. *Nature* 361, 642-644

8. Joyce, S., Tabaczewski, P., Angeletti, R. H., Nathenson, S. G., and Stroynowski, I. (1994) A nonpolymorphic major histocompatibility complex class Ib molecule binds a large array of diverse self-peptides. *J Exp Med* 179, 579-588

9. Tabaczewski, P., Chiang, E., Henson, M., and Stroynowski, I. (1997) Alternative peptide binding motifs of Qa-2 class Ib molecules define rules for binding of self and nonself peptides. *J Immunol* 159, 2771-2781

10. He, X., Tabaczewski, P., Ho, J., Stroynowski, I., and Garcia, K. C. (2001) Promiscuous antigen presentation by the nonclassical MHC Ib Qa-2 is enabled by a shallow, hydrophobic groove and self-stabilized peptide conformation. *Structure* 9, 1213-1224

11. Sherman, B. T., Hao, M., Qiu, J., Jiao, X., Baseler, M. W., Lane, H. C., Imamichi, T., and Chang, W. (2022) DAVID: a web server for functional enrichment analysis and functional annotation of gene lists (2021 update). *Nucleic Acids Res* 50, W216-W221
